# *S*-nitrosylation-dependent proteasomal degradation restrains Cdk5 activity to regulate hippocampal synaptic strength

**DOI:** 10.1038/ncomms9665

**Published:** 2015-10-27

**Authors:** Peng Zhang, Wing-Yu Fu, Amy K. Y. Fu, Nancy Y. Ip

**Affiliations:** 1 Divison of Life Science, The Hong Kong University of Science and Technology, Hong Kong, China; 2 Molecular Neuroscience Center, The Hong Kong University of Science and Technology, Hong Kong, China; 3 State Key Laboratory of Molecular Neuroscience, The Hong Kong University of Science and Technology, Hong Kong, China

## Abstract

Precise regulation of synaptic strength requires coordinated activity and functions of synaptic proteins, which is controlled by a variety of post-translational modification. Here we report that *S*-nitrosylation of p35, the activator of cyclin-dependent kinase 5 (Cdk5), by nitric oxide (NO) is important for the regulation of excitatory synaptic strength. While blockade of NO signalling results in structural and functional synaptic deficits as indicated by reduced mature dendritic spine density and surface expression of glutamate receptor subunits, phosphorylation of numerous synaptic substrates of Cdk5 and its activity are aberrantly upregulated following reduced NO production. The results show that the NO-induced reduction in Cdk5 activity is mediated by *S*-nitrosylation of p35, resulting in its ubiquitination and degradation by the E3 ligase PJA2. Silencing p35 protein in hippocampal neurons partially rescues the NO blockade-induced synaptic deficits. These findings collectively demonstrate that p35 *S*-nitrosylation by NO signalling is critical for regulating hippocampal synaptic strength.

## Abstract (web summary)

Phosphorylation of synaptic substrates by Cdk5 plays an essential role in synapse development. Here Zhang *et al.* reveal that *S*-nitrosylation of the activator of Cdk5, p35, by nitric oxide results in its degradation and reduced Cdk5 activity, leading to alterations in synaptic strength.

## Graphical abstract

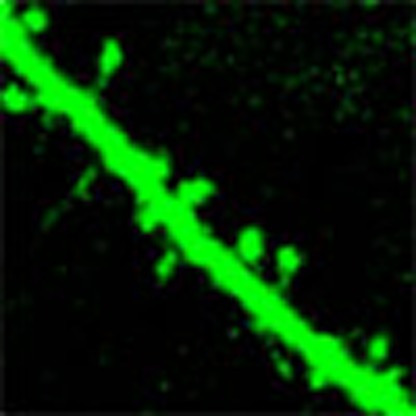

Precise regulation of structural and functional synaptic integrity is critical for neuronal network connectivity and proper brain functions. The structural and functional changes of excitatory synapses are often accompanied by changes in dendritic spine number, shape and neurotransmitter receptor contents^1^,^2^,^3^. These events are precisely regulated during synapse development, as well as subsequent plasticity in the adult brain. Abnormal dendritic spine morphology or surface abundance of neurotransmitter receptors is often associated with nervous system disorders, such as mental retardation, schizophrenia, autism and Alzheimer's disease^4^,^5^,^6^,^7^,^8^,^9^,^10^.

Accumulating evidence indicates that cyclin-dependent kinase 5 (Cdk5), a proline-directed serine/threonine kinase, plays an essential role in synapse development and functions^11^,^12^. While Cdk5 is activated through its association with the neuronal-specific activators p35 and p39 (ref. 13), its precise regulation is important for its functions at synapses. Intriguingly, Cdk5 can play either a positive or negative role in synapse development and functions; this is highly dependent on the phosphorylation of specific substrates. For example, the Cdk5-dependent phosphorylation of tropomyosin receptor kinase B (TrkB) in response to brain-derived neurotrophic factor is important for promoting the formation of dendritic spines in hippocampal neurons^14^. Regarding the inhibitory role of Cdk5 at synapses, Cdk5-dependent phosphorylation of the Rho guanine nucleotide exchange factor ephexin1 or the Wiskott–Aldrich syndrome protein family verprolin homologous protein 1 (WAVE1) reduces the number of dendritic spines through the modulation of actin dynamics^15^,^16^. Furthermore, Cdk5 negatively regulates the surface expression of *N*-methyl-D-aspartate (NMDA) glutamate receptor subunit NR2B through the phosphorylation of the postsynaptic protein PSD-95 (ref. 17) or NR2B itself^18^. Dysregulation of these processes, particularly Cdk5 hyperactivation, results in synaptic dysfunction, which is implicated in the pathogenesis of neurodegenerative diseases and psychiatric disorders^12^,^19^. These findings underscore the importance of the precise regulation of Cdk5 activity in the maintenance of synaptic integrity. Nonetheless, how Cdk5 activity and substrate phosphorylation are regulated in synapse maturation is unclear. The binding of Cdk5 to cyclin E was recently suggested to sequester Cdk5 to an inactive complex at synapses^20^, whereas the post-translational modification, that is, *S*-nitrosylation of Cdk5 at Cys83, reduces its kinase activity^21^.

Protein *S*-nitrosylation is a major post-translational modification that involves the reversible coupling of the gaseous intercellular messenger nitric oxide (NO) to cysteine residues; it plays well-characterized roles in synapse development and synaptic strength modulation^22^,^23^,^24^,^25^,^26^,^27^,^28^. For example, *S*-nitrosylation of PSD-95 regulates its targeting to synapses and hence its functions^29^, whereas stargazin *S*-nitrosylation enhances surface expression of α-amino-3-hydroxy-5-methyl-4-isoxazolepropionic acid (AMPA) glutamate receptors^29^,^30^. Whereas we previously identified Cdk5 as a substrate for *S*-nitrosylation^21^, it is unclear if there is any crosstalk between NO signalling and Cdk5 in synaptic development and plasticity. In this regard, it is noteworthy that glutamatergic excitatory neurotransmission inhibits Cdk5 activity^31^; meanwhile, excitatory neurotransmission can trigger neuronal nitric oxide synthase (nNOS) activation, resulting in NO production and protein *S*-nitrosylation^32^. Therefore, it is of great interest to determine whether NO regulates the development and functions at excitatory synapses through the modulation of Cdk5 activity.

In the present study, we report that NO signalling is important for both dendritic spine morphogenesis and glutamate receptor subunit surface expression in the adult mouse hippocampus. Furthermore, NO signalling negatively regulates Cdk5 activity through the *S*-nitrosylation of p35, resulting in its ubiquitin/proteasome-dependent degradation. The nNOS-knockout mouse hippocampus exhibits increased activity of Cdk5 and hyperphosphorylation of its substrates at synapses. The short hairpin RNA (shRNA)-mediated knockdown of p35 in NO signalling-deficient hippocampal neurons rescues the synaptic defects. These findings collectively reveal a novel crosstalk between NO signalling and Cdk5/p35 in which *S*-nitrosylation-dependent p35 degradation and suppression of Cdk5 activity are essential for the maintenance of excitatory synaptic strength.

## Results

### NO regulates synaptic strength in the adult hippocampus

**Figure 1:**
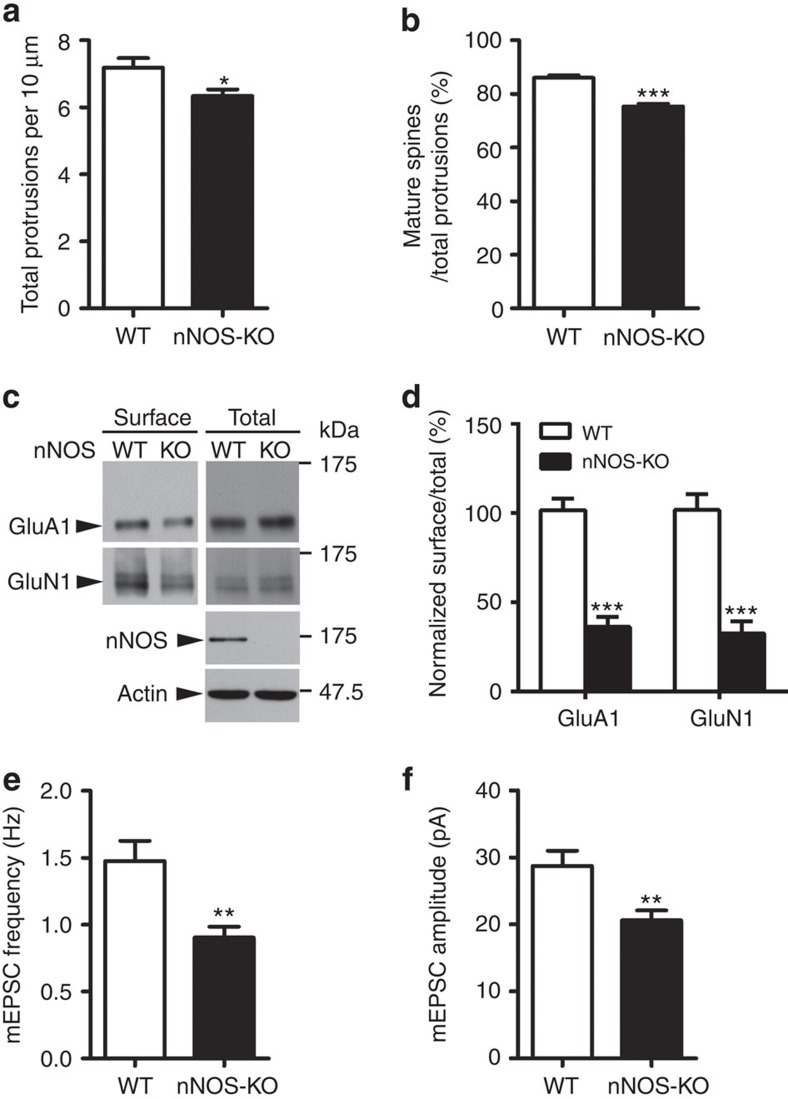
Compromised synaptic functions in nNOS-knockout (KO) hippocampal neurons. (**a**,**b**) nNOS-KO mice exhibited decreased spine density and percentages of mature spines. Quantification of dendritic spine density (**a**) and percentage of mature spines (**b**) in CA1 hippocampal pyramidal neurons from 3-month-old WT and nNOS-KO mice. Data represent the mean±s.e.m. of four independent experiments; *n*=30–32 secondary apical dendrites from four mice. *P=0.02, ***P=3.79E−10, unpaired Student's *t*-test. (**c**) nNOS-KO mice exhibited reduced GluA1 and GluN1 surface expressions. The surface proteins were analysed by biotin pulldown analysis. (**d**) Quantification of surface protein levels (percentage of surface expression to total) in WT and nNOS-KO mice. Data are normalized to WT control values and represent the mean±s.e.m. of four independent experiments, *n*=6 mice per condition. ***P=2.04E−5 (GluA1), ***P=1.34E−4 (GluN1); unpaired Student's *t*-test. (**e**,**f**) The mEPSC frequency and amplitude were decreased in nNOS-KO hippocampal neurons. Quantification of mEPSC frequency (**e**) and amplitude (**f**). Data represent the mean±s.e.m. of four independent experiments, *n*=15–20 neurons per condition. **P=0.0058 (frequency), **P=0.0047 (amplitude); unpaired Student's *t*-test.

To explore the effect of NO signalling on the structural and functional regulation of hippocampal synapses, we first examined dendritic spine morphology and density in green fluorescent protein (GFP)-expressing pyramidal neurons from the hippocampal CA1 region of 3-month-old wild-type (WT) and *nNOS*^−/−^ mice^33^. Genetic depletion of nNOS significantly decreased spine density and the percentage of mature spines (Fig. 1a,b; Supplementary Fig. 1a), suggesting a reduction of excitatory synapses. In addition to regulating spine density and morphology, NO signalling has been suggested to regulate the surface trafficking of AMPA receptors in cultured neurons^30^,^34^. Indeed, the surface expressions of both AMPA receptor subunit GluA1 and NMDA receptor subunit GluN1 were reduced in the adult *nNOS*^−/−^ mouse hippocampus (Fig. 1c,d; Supplementary Fig. 1b,c). These results demonstrate the importance of NO signalling for the maintenance of structural and functional synaptic strength and corroborate the finding that the absence of nNOS in the mouse hippocampus leads to molecular synaptic abnormalities.

Next, we investigated whether NO signalling influences excitatory neurotransmission in addition to its observed morphological and molecular effects. Interestingly, compared with WT neurons, the frequency of miniature excitatory postsynaptic currents (mEPSCs) was substantially reduced by ∼40% in cultured *nNOS*^−/−^ hippocampal neurons (Fig. 1e; Supplementary Fig. 1d). In addition, *nNOS*^−/−^ neurons exhibited reduced mEPSC amplitude (∼25%; Fig. 1f). To confirm that NO is indeed the direct mediator of these effects, we treated cultured rat hippocampal neurons with the nNOS inhibitor, L-NG-nitroarginine methyl ester (L-NAME); the resultant blockade of NO production had a similar effect (Supplementary Fig. 1e–g). These results collectively demonstrate that blocking endogenous NO production in hippocampal neurons reduces the number of excitatory synapses, consequently compromising excitatory neurotransmission.

### NO negatively regulates p35 levels and Cdk5 activity

**Figure 2:**
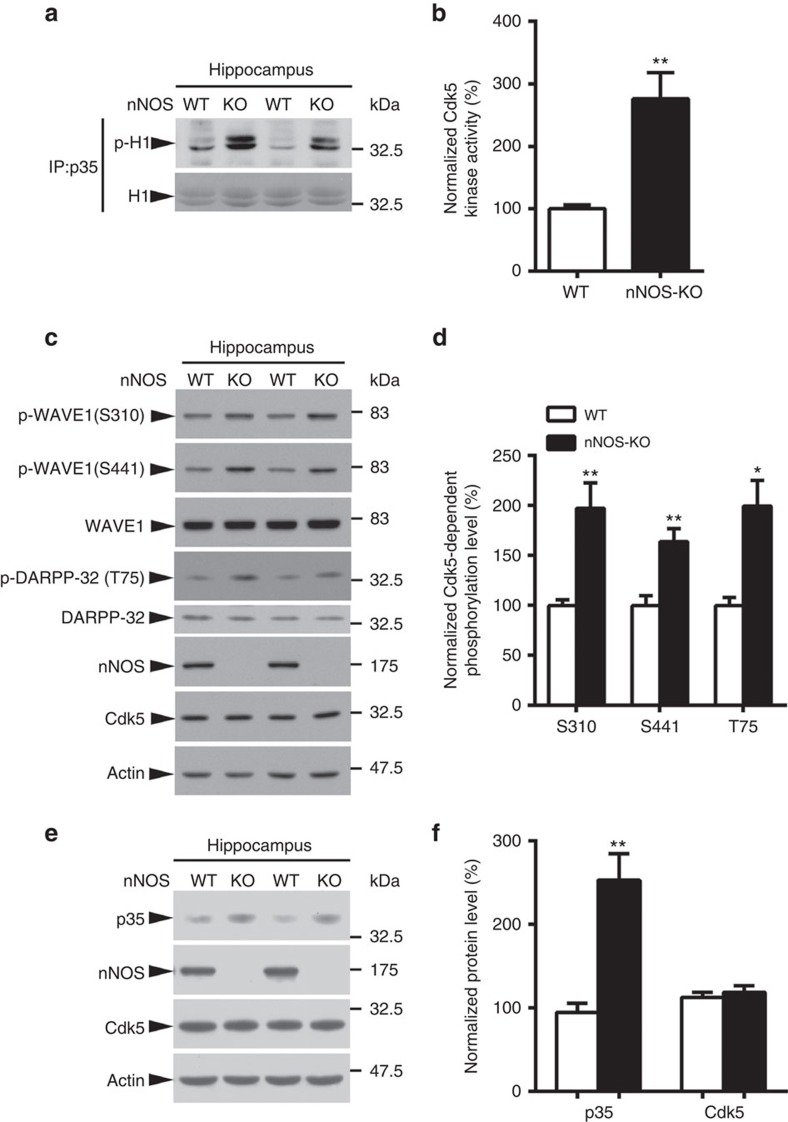
Increased Cdk5 activity and p35 level in the nNOS-knockout (KO) mouse hippocampus. (**a**,**b**) nNOS-KO mice exhibited enhanced Cdk5 activity in the hippocampus. (**a**) Hippocampal lysates of 3-month-old WT or nNOS-KO mice were subjected to the Cdk5 activity assay. (**b**) Percentage change of the kinase activity of Cdk5/p35 protein complex. Data are normalized to WT values and represent the mean±s.e.m. of four independent experiments, *n*=6 mice per condition. **P=0.0084; unpaired Student's *t*-test. (**c**,**d**) Cdk5-dependent phosphorylation of WAVE1 and DARPP-32 was increased in the nNOS-KO mouse hippocampus. (**c**) Hippocampal lysates of 3-month-old WT or nNOS-KO mice were subjected to western blot analysis using the indicated phospho-specific antibodies against the Cdk5 substrates WAVE1 and DARPP-32. (**d**) Quantification of phosphorylated protein levels (percentage of phosphorylated protein to total) in WT and nNOS-KO mice. Data are normalized to WT controls and represent the mean±s.e.m. of four independent experiments, *n*=6–8 mice per condition. **P=0.0025 (S310), **P=0.0018 (S441), *P=0.018 (T75); unpaired Student's *t*-test. (**e**,**f**) p35 was increased in the nNOS-KO mouse hippocampus. (**e**) Hippocampal lysates of 3-month-old WT or nNOS-KO mice were subjected to western blot analysis using the indicated antibodies. (**f**) Quantification of protein levels (ratio of specific protein to actin) in WT and nNOS-KO mice. Data are normalized to WT controls and represent the mean±s.e.m. of four independent experiments, *n*=6–8 mice per condition. **P=0.0055; unpaired Student's *t*-test.

We subsequently investigated the underlying molecular mechanism by which NO signalling modulates synaptic efficacy. Our laboratory previously found that NO suppresses Cdk5 activity under basal conditions via protein *S*-nitrosylation and that blockade of NO production leads to Cdk5 hyperactivation^21^. Concordantly, in the present study, the *in vitro* kinase assay revealed that *nNOS*^−/−^ mice exhibited enhanced hippocampal Cdk5 activity (Fig. 2a,b). WAVE1 and dopamine- and cAMP-regulated phosphoprotein of Mr 32,000 (DARPP-32) are well-known modulators of synaptic functions; Cdk5-dependent phosphorylation inhibits their activities to regulate dendritic spines and glutamatergic receptor surface expression^16^,^35^, respectively. Therefore, we determined whether *nNOS* elimination alters the Cdk5-dependent phosphorylation status of these two synaptic proteins in the brain. As expected, *nNOS*^−/−^ mice exhibited increased WAVE1 and DARPP-32 phosphorylation in the hippocampus (Fig. 2c,d), suggesting that the deregulation of Cdk5 and its synaptic substrates might contribute to synaptic failure on NO production blockade.

Unexpectedly, p35 protein expression was elevated in the *nNOS*^−/−^ mouse hippocampus (Fig. 2e,f). This raised the intriguing possibility that in addition to directly *S*-nitrosylating Cdk5 and inhibiting its activity^21^, NO might also control p35 protein level to constrain Cdk5 activity. Therefore, we determined whether endogenous NO controls p35 protein level. Silencing nNOS increased p35 expression; conversely, ectopic nNOS overexpression reduced p35 level in cultured rat cortical neurons (Supplementary Fig. 2a–d). Furthermore, we treated cultured rat cortical neurons with the nNOS inhibitor, L-NAME; the resultant blockade of NO production resulted in an obvious increase of p35 level (Supplementary Fig. 2e–g), as well as the Cdk5-dependent phosphorylation of WAVE1 (Supplementary Fig. 2g). These results collectively indicate that NO negatively regulates p35 protein level and its associated Cdk5 activity, and that blocking NO production leads to Cdk5 hyperactivation in the adult mouse hippocampus.

### *S*-nitrosylation of p35 promotes its degradation

**Figure 3:**
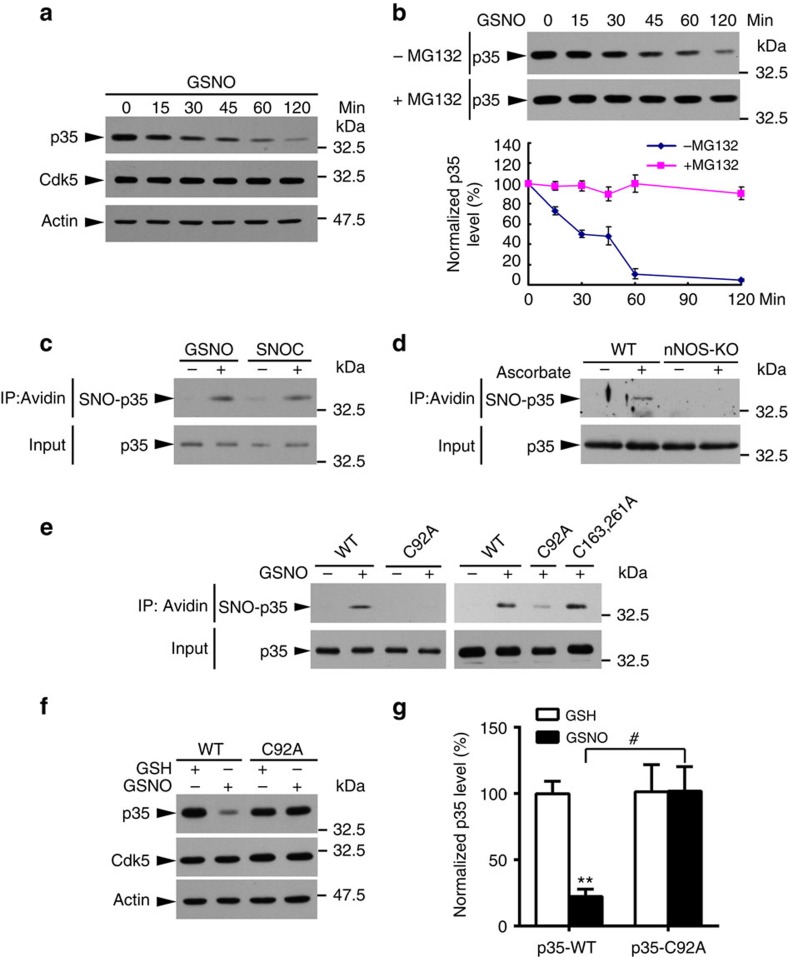
p35 *S*-nitrosylation at Cys92 promotes its degradation. (**a**) GSNO treatment decreased p35 protein level. Cultured rat cortical neurons at 8–10 days *in vitro* were treated with GSNO for the indicated times and then subjected to western blot analysis. (**b**) GSNO decreased p35 level protein in a proteasome-dependent manner. Cultured rat cortical neurons were treated with GSNO in the presence or absence of the proteasome inhibitor MG132 for the indicated times and then subjected to western blot analysis for p35 protein. Representative blots (top) and quantification (bottom). (**c**) NO *S*-nitrosylated p35 *in vitro*. Lysates of HEK293T cells overexpressing p35 were incubated with NO donor GSNO or SNOC for 30 min and subsequently subjected to the biotin switch assay. (**d**) p35 was *S*-nitrosylated in the adult mouse brain. Brain lysates of adult WT and nNOS-knockout (KO) mice were subjected to the biotin switch assay in the presence or absence of ascorbate as indicated. (**e**) Cys92 was the target site for p35 *S*-nitrosylation. HEK293T cells were transfected with expression constructs encoding the WT or specific cysteine mutants of p35 as indicated. The cell lysate was then incubated with GSNO for 30 min and subjected to the biotin switch assay. The biotinylated proteins were immunoprecipitated (IP) with NeutrAvidin agarose followed by western blot analysis for p35. (**f**,**g**) Cys92 *S*-nitrosylation of p35 promoted its degradation. (**f**) COS7 cells were transfected with p35-WT or p35-C92A mutant; 24 h after transfection, cells were treated with GSH/GSNO in the presence of doxycycline (Dox) for 6 h and subjected to western blot analysis. (**g**) Quantification of p35 level (relative to the actin level). Data are normalized to control treatment and represent mean±s.e.m. from four independent experiments. **P=0.001, ^#^*P*=0.019; one-way analysis of variance with the Student–Newman–Keuls test.

To confirm that NO directly controls p35 protein level, cultured rat cortical neurons were treated with the physiological NO donor, *S*-nitrosoglutathione (GSNO); this markedly reduced p35 level in a time-dependent manner (Fig. 3a). The resultant protein reduction could be due to either the inhibition of protein synthesis or enhancement of protein degradation. To determine which mechanism is responsible for the observed effect, we compared the degradation rate of p35 in cultured rat cortical neurons treated with GSNO or the protein synthesis inhibitor cycloheximide (CHX). Because p35 level decreased much faster in neurons treated with GSNO than with CHX (Supplementary Fig. 3a), this suggests that the effect of NO signalling is mediated at least in part through enhanced p35 degradation. To confirm this, cultured rat cortical neurons were treated with GSNO together with a proteasome inhibitor, MG132 which completely blocked the GSNO-induced reduction of p35 levels (Fig. 3b), indicating that the NO-induced rapid reduction of p35 occurs via proteasome activity.

NO has been suggested to *S*-nitrosylate free cysteines in some proteins, modulating their stability^36^,^37^,^38^. Moreover, p35 is a labile protein, whose expression level changes markedly with respect to neuronal activity^31^,^39^. Therefore, NO may regulate p35 protein level via *S*-nitrosylation. We first evaluated whether p35 can be *S*-nitrosylated by NO donors. To this end, HEK293T cell lysate overexpressing p35 was incubated with the physiological NO donors, GSNO or *S*-nitrosocysteine (SNOC), which readily *S*-nitrosylate p35 (Fig. 3c). In particular, ascorbate-dependent basal *S*-nitrosylation of endogenous p35 was detected in WT but not *nNOS*^−/−^ mouse brains; this suggests that p35 is *S*-nitrosylated *in vivo* in a nNOS-dependent manner (Fig. 3d). Furthermore, neuronal activity (for example, NMDAR activation) increased p35 *S*-nitrosylation in cultured rat cortical neurons (Supplementary Fig. 3b), indicating the dynamic property of this post-translational modification. Moreover, treatment of cultured rat cortical neurons with the reducing reagent dithiothreitol (DTT), which abolished protein *S*-nitrosylation, increased Cdk5 activity (Supplementary Fig. 3c), confirming the inhibitory role of protein *S*-nitrosylation on Cdk5 activity.

*S*-nitrosylation occurs at cysteine residues^40^. To identify the *S*-nitrosylation site(s) of p35, we first analysed its amino-acid sequence, which revealed the presence of eight cysteine residues (Supplementary Fig. 3d). Accordingly, we generated mutants in which each of the eight cysteine residues or combinations thereof were substituted with alanine, which cannot be *S*-nitrosylated. HEK293T cells were transfected with p35-WT or its cysteine-to-alanine (C→A) mutants, and the cell lysates were subsequently subjected to the biotin switch assay. Incubating the cell lysate with GSNO robustly *S*-nitrosylated p35-WT. In contrast, expression of the C92A mutant alone drastically reduced p35 *S*-nitrosylation, suggesting that Cys92 is the major *S*-nitrosylation site. Other cysteine mutants of p35 were *S*-nitrosylated to a similar extent as p35-WT, corroborating the notion that Cys92 is the single major *S*-nitrosylation site on p35 (Fig. 3e; Supplementary Fig. 3e).

Next, we determined whether p35 *S*-nitrosylation at Cys92 mediates its GSNO-induced degradation. To this end, p35-WT or its *S*-nitrosylation-deficient mutant p35-C92A was overexpressed in COS7 cells using an inducible Tet^OFF^ system (Supplementary Fig. 3f). While p35 was strongly expressed in the absence of doxycycline, the addition of doxycycline, which inhibits p35 protein synthesis, resulted in a rapid loss of p35 within 4 h (Supplementary Fig. 3g). Furthermore, the addition of GSNO accelerated the rate of p35 protein turnover; however, this effect was abolished for p35 mutant lacking the C92 *S*-nitrosylation site (Fig. 3f,g).

**Figure 4:**
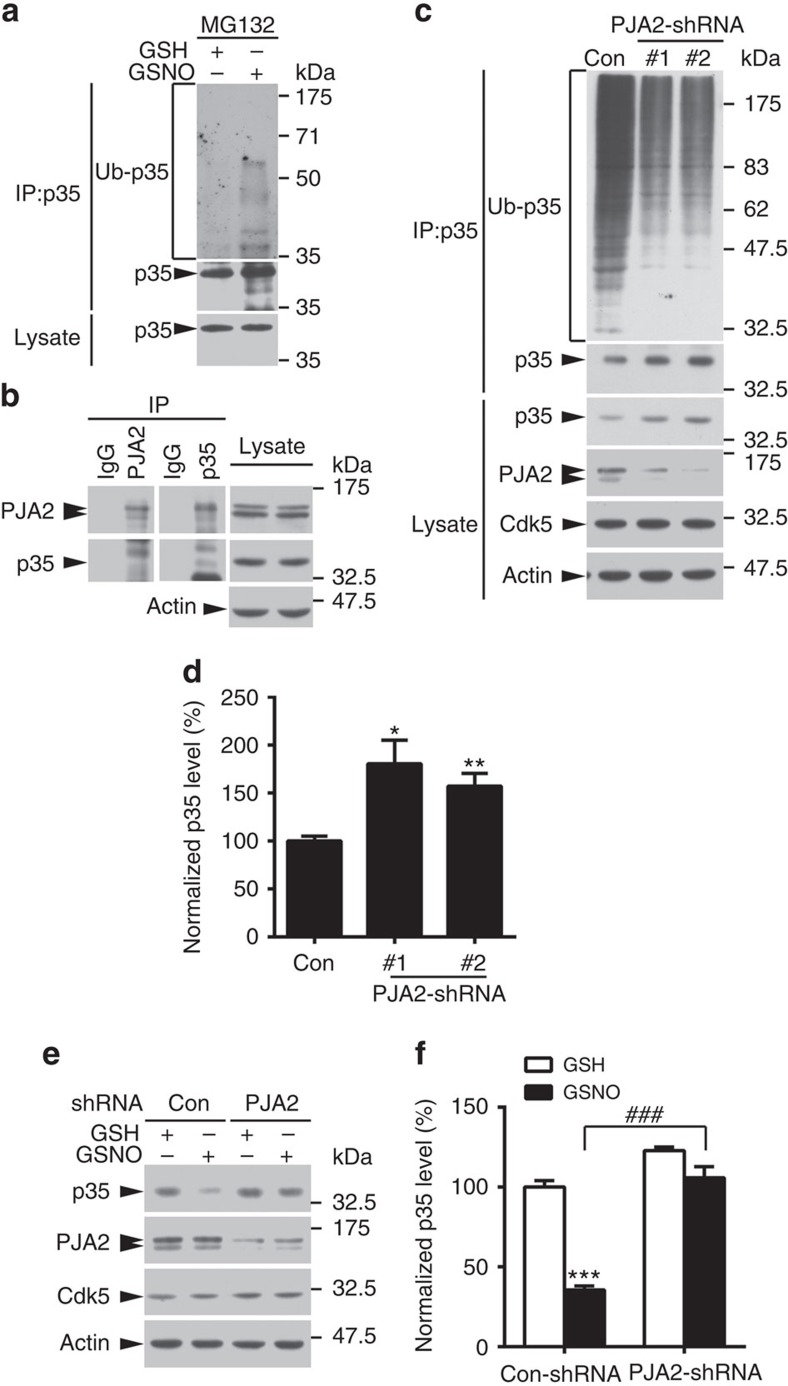
p35 degradation in neurons is mediated through the E3 ubiquitin ligase PJA2. (**a**) GSNO treatment increased p35 polyubiquitination level. Cultured rat cortical neurons were treated with GSNO in the presence of the proteasome inhibitor MG132. The protein lysate was subjected to immunoprecipitation using p35 antibody followed by western blot analysis for polyubiquitin. (**b**) p35 associated with PJA2 in the mouse hippocampus. Hippocampal lysates of 3-month-old mice were immunoprecipitated (IP) using p35 or PJA2 antibodies (IgG as control) as indicated, and the proteins in the co-immunoprecipitates were examined by western blot analysis. (**c**,**d**) p35 degradation is mediated by the E3 ligase PJA2. (**c**) PJA2 was knocked down in cultured rat cortical neurons, and the endogenous expression of p35 was examined by western blot analysis. (**d**) Quantification of p35 protein level (relative to the actin level). Data are normalized to the control and represent the mean±s.e.m. of four independent experiments. *P=0.016, **P=0.0018; one-way analysis of variance (ANOVA) with the Student–Newman–Keuls test. (**e**,**f**) GSNO promotes p35 degradation in a PJA2-dependent manner. (**e**) PJA2 was knocked down with PJA2-shRNA (#1) in cultured rat cortical neurons for 4 days, the cells were treated with GSH/GSNO for 1 h and p35 protein level was examined by western blot analysis. (**f**) Quantification of p35 level (relative to the actin level). Data are normalized to control group and represent the mean±s.e.m. of six independent experiments. ***P=6.81E−7, ^###^*P*=6.42E−5; one-way ANOVA with the Student–Newman–Keuls test.

We also detected increased polyubiquitination of p35 after GSNO treatment (Fig. 4a), suggesting that NO signalling reduces p35 abundance in a ubiquitin/proteasome-dependent manner. The recent identification of Praja-2 (PJA2) as the E3 ligase of p35 in pancreatic β-cells^41^ prompted us to investigate whether the NO-induced degradation of p35 in the nervous system is also mediated by PJA2. Interestingly, PJA2 and p35 were detected in a protein complex in mouse hippocampal lysate (Fig. 4b). Silencing PJA2 expression in cultured rat cortical neurons reduced p35 polyubiquitination and resulted in p35 accumulation (Fig. 4c,d). Moreover, suppressing endogenous PJA2 expression by shRNA knockdown blocked GSNO-induced p35 degradation (Fig. 4e,f). These findings indicate that NO reduces p35 abundance by promoting its ubiquitination by the E3 ligase PJA2. Moreover, when cells overexpressing p35-WT or p35-C92A were treated with GSNO, polyubiquitination and binding with PJA2 were enhanced in p35-WT cells, while neither was observed in p35-C92A cells (Supplementary Fig. 4). These results collectively indicate that p35 *S*-nitrosylation at Cys92 specifically promotes its ubiquitin/proteasome-dependent degradation in a PJA2-dependent manner.

### Cdk5 inhibition reverses spine defects in *nNOS* ^−/−^ neurons

**Figure 5:**
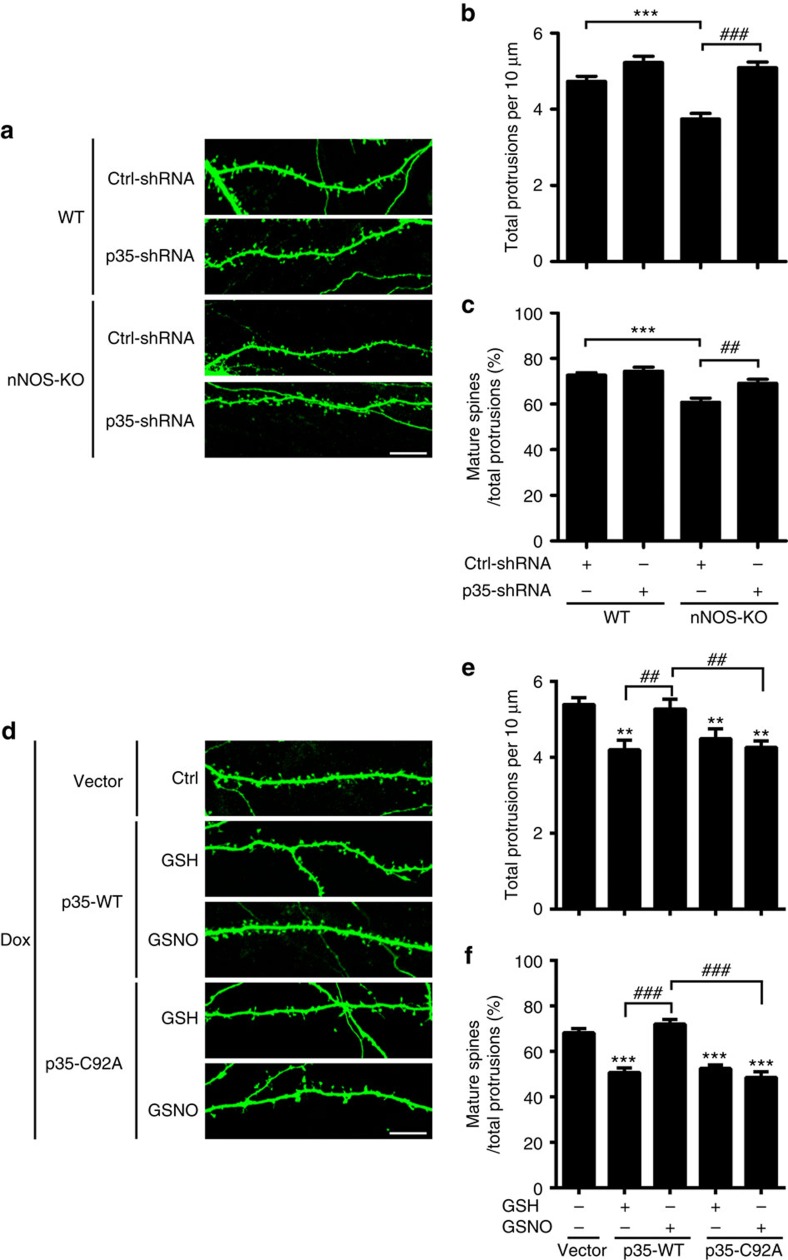
NO signalling regulates spine density and morphology via p35 *S*-nitrosylation at Cys92 and downregulation of Cdk5 activity. (**a**–**c**) NO signalling regulated spine density and morphology in a p35-dependent manner. Hippocampal neurons derived from WT or nNOS-knockout (KO) mice were transfected with control (Ctrl)-shRNA or p35-shRNA together with a GFP construct at 12–13 days *in vitro* and fixed at 20 days *in vitro*. Representative images (**a**) are shown. Scale bar, 10 μm. Quantification of spine density (**b**) and percentages of mature spines (**c**). Data represent the mean±s.e.m. of four independent experiments; *n*=24–30 dendrites from 10–15 neurons. Total protrusions: ***P=6.03E−6, ^###^*P*=2.68E−8; mature spine proportion: ***P=2.39E−6, ^##^*P*=0.002; one-way analysis of variance (ANOVA) with the Student–Newman–Keuls test. (**d**–**f**) *S*-nitrosylation of p35 at Cys92 regulated spine density and morphology. Cultured rat hippocampal neurons at 15–16 days *in vitro* (DIV) were transfected with a cDNA construct encoding p35-WT or p35-C92A using an inducible Tet^OFF^ system; neurons at 20–22 DIV were then treated with GSH/GSNO (50 μM) for 8 h in the presence of doxycycline (Dox). (**d**) Representative images are shown. Scale bar, 10 μm. Quantification of spine density (**e**) and percentages of mature spines (**f**). Data represent the mean±s.e.m. of four independent experiments; *n*=15–21 dendrites from 8–10 neurons. Total protrusions: **P=0.0037 (p35-WT+GSH), **P=0.0065 (p35-C92A+GSH), **P=0.0058 (p35-C92A+GSNO), ^##^*P*=0.0045 (p35-WT+GSH versus p35-WT+GSNO), ^##^*P*=0.0024 (p35-WT+GSNO versus p35-C92A+GSNO); mature spine proportion: ***P=4.56E−7 (p35-WT+GSH), ***P=8.81E−7 (p35-C92A+GSH), ***P=3.14E−7 (p35-C92A+GSNO), ^###^*P*=5.59E−9 (p35-WT+GSH versus p35-WT+GSNO), ^###^*P*=6.33E−9 (p35-WT+GSNO versus p35-C92A+GSNO); one-way ANOVA with the Student–Newman–Keuls test.

Cdk5 negatively regulates the morphogenesis of dendritic spines^15^,^20^ where excitatory synapses reside. Given the abnormal p35 accumulation and Cdk5 hyperactivation in the *nNOS*^−/−^ mouse hippocampus, we hypothesized that reducing the Cdk5/p35 activity would rescue the synaptic deficits observed on NO production blockade. To test this hypothesis, we silenced p35 expression in cultured hippocampal neurons from WT and *nNOS*^−/−^ mice. Quantitative analysis showed that the density of total protrusions and percentage of mature spines decreased significantly in *nNOS*^−/−^ neurons; meanwhile, the inhibition of Cdk5 activity by p35-shRNA rescued the spine deficits of *nNOS*^−/−^ neurons (Fig. 5a–c). To substantiate these results, cultured rat hippocampal neurons were treated with L-NAME in the presence or absence of the specific Cdk5 inhibitor roscovitine. L-NAME decreased both the density of total protrusions and percentage of mature spines, while Cdk5 inhibition rescued these deficits (Supplementary Fig. 5a–c). These results collectively suggest that Cdk5 hyperactivation is at least partially responsible for the spine defects observed in hippocampal neurons when NO production is impaired.

### p35 *S*-nitrosylation at Cys92 regulates synaptic strength

Given that p35 *S*-nitrosylation at Cys92 regulates its degradation and that p35 loss can influence spine morphogenesis, we subsequently determined whether p35 *S*-nitrosylation at Cys92 specifically regulates dendritic spine morphology. Either p35-WT or its C92A mutant was overexpressed in cultured rat hippocampal neurons using an inducible Tet^OFF^ system. p35-WT overexpression reduced dendritic spine density and maturation, while GSNO treatment abolished this effect. However, GSNO treatment did not affect spine number or maturation in p35-C92A-expressing neurons (Fig. 5d–f), indicating that NO regulates dendritic spine morphology and density through p35 *S*-nitrosylation and hence the modification of Cdk5 activity.

**Figure 6:**
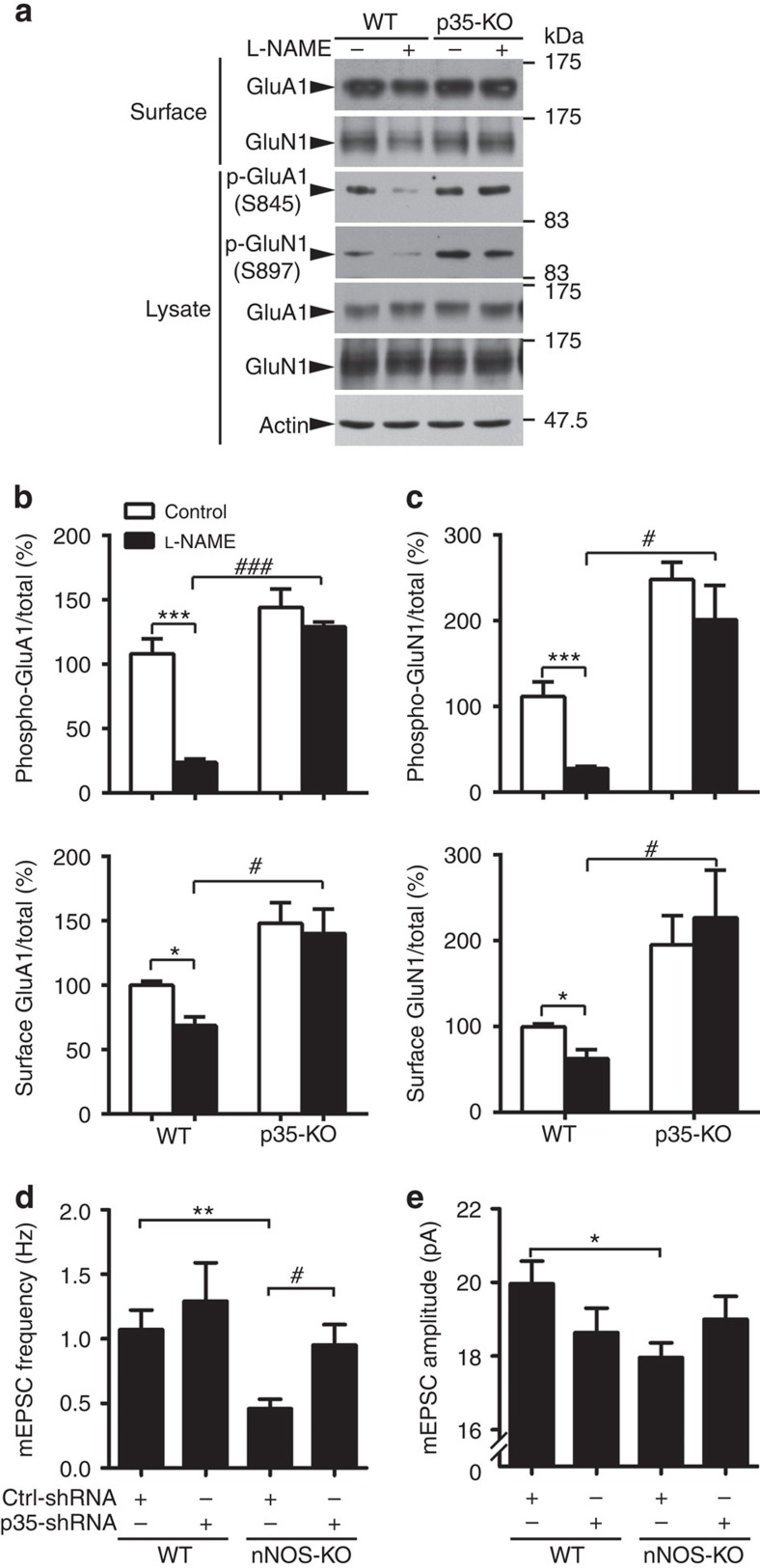
p35 *S*-nitrosylation regulates GluA1 and GluN1 surface expressions and excitatory neurotransmission. (**a**–**c**) NO signalling regulates GluA1 and GluN1 surface expressions in a p35-dependent manner. (**a**) Acute brain slices from 2–3-month-old WT or p35-knockout (KO) mice were treated with L-NAME for 4 h and subsequently subjected to the surface biotinylation assay. (**b**,**c**) Quantification of phospho and surface protein levels (ratio of phospho and surface expression to total) in WT and p35-KO mouse hippocampal slices. Data are normalized to the WT control group and represent the mean±s.e.m. of four independent experiments, *n*=6 mice per condition. Phospho-GluA1: ***P=0.00038, ^###^*P*=4.87E−7 (WT+L-NAME versus p35-KO+L-NAME); phospho-GluN1: ***P=0.00027, ^#^*P*=0.022 (WT+L-NAME versus p35-KO+L-NAME); surface GluA1: *P=0.039, ^#^*P*=0.011 (WT+L-NAME versus p35-KO+L-NAME); surface GluN1: *P=0.014, ^#^*P*=0.031 (WT+L-NAME versus p35-KO+L-NAME); one-way analysis of variance (ANOVA) with the Student–Newman–Keuls test. (**d**,**e**) Hippocampal neurons derived from WT or nNOS-KO mice were transfected with Ctrl-shRNA or p35-shRNA together with GFP construct at 12–13 days *in vitro* (DIV), and mEPSCs were recorded at 20 DIV. Quantification of frequency (**d**) and amplitude (**e**) of mEPSCs. Data represent the mean±s.e.m. of four independent experiments, *n*=12–15 neurons per condition. **P=0.0091,*P=0.048, ^#^*P*=0.043; one-way ANOVA with the Student–Newman–Keuls test.

The cAMP-dependent protein kinase (PKA)-mediated phosphorylation of GluA1 and GluN1 is required for their surface expressions, as well as synaptic strength maintenance. Interestingly, Cdk5 activity can inhibit GluA1 and GluN1 phosphorylation at Ser845 and Ser897, respectively, via the Cdk5/DARPP-32/PKA cascade^20^,^42^. Given the association between Cdk5 hyperactivation and reduced GluA1 and GluN1 surface expressions triggered by NO production blockade, elevated p35 and the resultant Cdk5 activity plausibly contribute to the synaptic dysfunctions observed on NO production blockade. Indeed, blocking NO production by L-NAME treatment reduced GluA1 and GluN1 phosphorylation and surface expressions in WT mouse hippocampal slices. Similar reduction was not observed in p35-knockout mouse hippocampal slices (Fig. 6a–c). L-NAME treatment also reduced GluA1 and GluN1 surface expression in cultured rat hippocampal neurons. The inhibition of Cdk5 activity by roscovitine abolished these reductions (Supplementary Fig. 6a–d). Therefore, the reduced surface expressions of GluA1 and GluN1 in the *nNOS*^−/−^ mouse hippocampus (Fig. 1c,d) are very likely mediated via p35 accumulation and Cdk5 hyperactivation.

To further elucidate the role of p35 *S*-nitrosylation at Cys92 in the regulation of surface GluA1, either p35 or p35-C92A together with GluA1 constructs was overexpressed in HEK293T cells and the surface level of GluA1 was examined. Overexpression of p35-WT or p35-C92A reduced GluA1 surface expression, which is consistent with the inhibitory role of Cdk5 on surface AMPA receptor trafficking. More importantly, GSNO treatment abolished the reduction of surface GluA1 in cells expressing p35-WT but not p35-C92A (Supplementary Fig. 6e,f). These findings suggest that p35 *S*-nitrosylation at Cys92 determines p35 protein levels and regulates GluA1 surface expression in a p35-dependent manner.

### Cdk5 inhibition reverses synaptic defects in *nNOS* ^−/−^ neurons

To confirm these findings at the functional level, we silenced p35 expression in cultured hippocampal neurons from WT and *nNOS*^−/−^ mice and performed electrophysiological studies. Quantitative analysis showed that inhibition of Cdk5 activity by p35-shRNA rescued the mEPSC frequency but not the amplitude deficits observed in *nNOS*^−/−^ neurons (Fig. 6d,e; Supplementary Fig. 7a,b). While mEPSC frequency is a measure of the probability of presynaptic glutamate neurotransmitter contacting postsynaptic AMPA receptors^43^, mEPSC amplitude indicates the synaptic size^44^. This may suggest that NO signalling regulates synapse number by modulating the protein level of p35 and affects the synapse size through an independent mechanism. Taken together, these findings indicate that p35 accumulation and Cdk5 hyperactivation contribute to the synaptic defects generated on NO production blockade and that inhibition of Cdk5 activity can at least partially rescue synaptic failure in *nNOS*^−/−^ neurons.

## Discussion

Whereas Cdk5/p35 is critical for the early development of the central nervous system^45^, p35 expression and Cdk5 activity are downregulated during the onset of synaptogenesis and synaptic maintenance in mouse and rat brains^46^. These findings suggest that proper synaptic integrity requires the precise spatiotemporal regulation of p35 protein level and Cdk5 activity. The identification of cyclin E as a competitor of Cdk5/p35 complex partially explains how Cdk5 activity is suppressed in synaptic function and memory formation^20^. Nonetheless, the molecular mechanism controlling p35 abundance in mouse/rat hippocampus during development remains largely unknown. The present study provides compelling evidence that NO signalling restricts Cdk5 activity through the p35 *S*-nitrosylation-dependent regulation of its protein abundance. NO production results in p35 *S*-nitrosylation, leading to its degradation in a PJA2-dependent manner. On the other hand, disruption of p35 degradation by NO production blockade as in *nNOS*^−/−^ mouse hippocampi or L-NAME-treated neurons prominently increases p35 level and Cdk5 activity accompanied by excitatory synaptic dysfunction. Taken together, these findings demonstrate that the diffusible gas NO acts as a molecular switch that controls dendritic spine morphogenesis, glutamate receptor surface expression and synaptic efficacy by restricting Cdk5 activity through the p35 *S*-nitrosylation-dependent manner (Supplementary Fig. 8).

Our findings reveal that NO signalling restricts Cdk5 activity through two distinct mechanisms. The first mechanism involves *S*-nitrosylation of Cdk5 at Cys83, which inhibits its kinase activity likely by perturbing the binding to ATP^21^. The second novel mechanism revealed in the present study is mediated by *S*-nitrosylation of p35, which results in its degradation and reduced protein abundance. Why does NO signalling controls Cdk5 activity in such a complex manner? One possible explanation is that Cdk5 activity requires strict spatiotemporal coordination to ensure proper synaptic development and subsequent plasticity. NO promptly *S*-nitrosylates Cdk5 within several minutes to inhibit Cdk5 kinase activity. However, like phosphorylation, protein *S*-nitrosylation is reversible and dynamic with a very short half-life, implying that the NO-dependent suppression of Cdk5 is transient. To achieve a sustained suppressive effect, NO *S*-nitrosylates p35 and controls its protein abundance by promoting its degradation. Consistent with this hypothesis, we previously observed that treating cortical neurons with a NO donor for a very short time (∼5–15 min) inhibited Cdk5 activity without altering p35 protein level^21^, while longer exposure significantly reduced p35 protein level accompanied by longer suppression of Cdk5 activity. Furthermore, *S*-nitrosylated Cdk5 can transfer the NO group to other proteins via a molecular mechanism termed ‘transnitrosylation'^47^. Given the physiological interaction between Cdk5 and p35, it is reasonable to speculate that SNO-Cdk5 *S*-nitrosylates p35 via transnitrosylation. Thus, it is of great interest to determine whether NO *S*-nitrosylates p35 and promotes its degradation in a Cdk5-dependent or Cdk5-independent manner. Our findings also indicate that p35 *S*-nitrosylation occurs at Cys92 and promotes its degradation by enhancing its binding to the E3 ligase PJA2. Of note, Sakamaki *et al.*^41^ report that salt-inducible kinase 2 phosphorylates p35 at Ser91 and promotes its ubiquitin-dependent degradation in pancreatic β-cells. This raises the interesting possibility that phosphorylation at Ser91 or *S*-nitrosylation at Cys92 may alter the protein structure of p35 and promotes its accessibility to the E3 ligase PJA2, thus targeting it to the proteasome system. Another outstanding question is whether the p35 *S*-nitrosylation at Cys92 directly affects its accessibility to PJA2 or alters this by regulating the p35 phosphorylation level at Ser91. Moreover, it is noteworthy that both Ser91 and Cys92 are located in the N-terminal p10 domain of p35 (ref. 45); this may partially explain why p35 protein has a relatively rapid turnover rate while p25 does not. Intriguingly, p39, another activator of Cdk5, can be *S*-nitrosylated under some conditions, specifically when the lysate of cells overexpressing p39 are treated with different NO donors (that is, GSNO or SNOC; data not shown). In addition, while Cys92 is not conserved in p39, two other candidate *S*-nitrosylation sites were identified in p39 (data not shown).

How does NO/Cdk5 signalling regulate the structural and functional properties of excitatory synapses? Under basal conditions, Cdk5/p35 may act as a ‘brake' to restrain synaptic strength through the inhibitory phosphorylation of specific synaptic substrates. For instance, the Cdk5 substrate WAVE1 promotes dendritic spine maturation via the regulation of actin dynamics, while Cdk5-dependent phosphorylation inhibits its activity, reducing mature spines and functional synapses^16^,^39^. In parallel, the PKA-dependent phosphorylation of glutamate receptor subunits GluA1 and GluN1 is important for their surface expressions, while the Cdk5-dependent phosphorylation of DARPP-32 inhibits PKA activity, which in turn reduces the phosphorylation levels and surface expressions of GluA1 and GluN1 (refs 20, 35, 42). Therefore, p35 is *S*-nitrosylated in response to NO production, and its expression level is downregulated; this is followed by the release of the Cdk5/p35 ‘brake,' which increases spine density and maturation as well as the surface expressions of AMPA and NMDA receptors. Therefore, we propose that NO signalling is critical for the maintenance of synaptic strength by restricting Cdk5 activity and precisely controlling the phosphorylation status of specific synaptic proteins (Supplementary Fig. 8). To better define the roles of NO signalling in spine morphogenesis, it will be interesting to investigate whether NO production results in enhanced formation of new spines, reduced elimination of pre-existing spines or both. In addition, whether NO production results in enhanced exocytosis or decreased endocytosis of GluA1, GluN1 or both requires further investigation.

NO is produced at excitatory synapses as a result of synaptic activation through the associations of nNOS with PSD-95 and NMDA receptors^32^. In the present study, nNOS expression was observed in different types of neurons including mouse hippocampal CA3-CA1 pyramidal neurons and GABAergic interneurons (data not shown). Nonetheless, how NO production is precisely controlled at synapses remains unclear. Moreover, it is noteworthy that glutamate-dependent neuronal stimulation and KCl-induced neuronal depolarization can trigger nNOS activation, resulting in NO production and protein *S*-nitrosylation^48^,^49^,^50^. Interestingly, excitatory glutamatergic neurotransmission and neuronal depolarization reduce Cdk5 activity by enhancing the proteasome-dependent degradation of p35 (refs 31, 39), making p35 *S*-nitrosylation a candidate mechanism for inducing protein degradation and subsequent changes in synaptic strength in response to glutamate/KCl-mediated neuronal activity. Therefore, it would be of interest to determine whether p35 *S*-nitrosylation plays a regulatory role in experiences such as enriched environments, fear conditioning and associative learning-dependent synaptic pruning or rearrangement *in vivo*.

The deregulation of Cdk5, particularly hyperactivation, is implicated in several psychiatric disorders and neurodegenerative diseases characterized by severely impaired cognitive functions^12^,^13^,^19^; accordingly, the inhibition of Cdk5 activity can alleviate the synaptic losses and cognitive impairments in certain pathological conditions^51^,^52^. These observations collectively raise the exciting possibility that the deregulation of the *S*-nitrosylation-dependent proteasomal degradation of p35 pathway may contribute to the synaptic impairment underlying the cognitive dysfunctions in diseased conditions.

## Methods

### Reagents and plasmids

All chemicals were purchased from Sigma unless stated otherwise. For the Tet^OFF^ system, the plasmid ratio for co-transfection was 1:10 (pUHD15.1:pTRE2)^53^. Modified pUHD15.1 and pTRE2 vectors were gifts from M. Chao (New York University, New York). The mouse p35 sequence was subcloned into the pTRE2 vector. The pSUPER-based shRNA construct for rat/mouse p35 with a target sequence of 5′-TATCAACCTCATGAGCTCC-3′, rat nNOS with a target sequence of 5′-GGGCCAAGAAAGTCTTCAA-3′ and rat PJA2 with target sequences of 5′-TGCCTGTFFAFAGTATTAC-3′ (#1) and 5′-TGCCTCAAGTGTCCAGAAT-3′ (#2) were constructed. The antibodies against phospho-WAVE1 (S310 & S441, 1:2,000) and total WAVE1 (1:5,000) were gifts from P. Greengard (Rockefeller University, New York). Additional primary antibodies included those against PSD-95 (1:1,000, MA1-046, Thermo Scientific), Cdk5 (1:1,000, DC17 & C8, Santa Cruz), p35 (1:1,000, C19, Santa Cruz), synaptophysin (1:10,000, MAB5258, Chemicon), phospho-Histone H1 (1:1,000, 32078, Upstate), phospho-S845-GluA1 (1:1,000, p1160-845, PhosphoSolutions), GluA1 (1:2,000, MAB2263, Millipore), phospho-S897-GluN1 (1:500, ABN99, Millipore), GluN1 (1:1,000, 556308, BD Pharmingen), polyubiquitin (1:1,000, FK2, Enzo Life Science) and actin (1:5,000, A3853, Sigma). Antibodies against p35 (1:1,000, 2680), nNOS (1:1,000, 4321), phospho-T75-DARPP-32 (1:1,000, 2301) and DARPP-32 (1:1,000, 2302) as well as horseradish peroxidase-conjugated goat anti-mouse and anti-rabbit antibodies were from Cell Signaling.

### nNOS transgenic mice and rats

All animal experiments were conducted in accordance with the Guidelines of the Animal Care Facility of HKUST and were approved by the Animal Ethics Committee at HKUST. The nNOS transgenic mice (002986) were obtained from the Jackson Laboratory.

### Site-directed mutagenesis

Site-directed mutagenesis was performed with oligonucleotide primers (Supplementary Table 1) designed to substitute the corresponding cysteine residue(s) with alanine residue(s) using the QuikChange site-directed mutagenesis kit (Stratagene) according to the manufacturer's instructions.

### Surface biotinylation assay

Cultured rat hippocampal neurons and acute mouse hippocampal slices were washed with ice-cold Dulbecco's phosphate buffer (DPBS) and subsequently incubated with 1 mg ml^−1^ EZ-link-sulfo-LC-Biotin and 0.5 mg ml^−1^ EZ-link-sulfo-SS-Biotin (Pierce) in DPBS for 30 min, respectively. The biotin solution was then removed, and the remaining unreacted biotin was quenched by the addition of 50 mM Tris solution (pH 7.4) for 2 min. The neurons and slices were then washed with ice-cold DPBS and lysed with radioimmunoprecipitation assay buffer supplemented with protease inhibitors. Biotinylated proteins were separated from other proteins using streptavidin agarose overnight at 4 °C. The beads were then washed three times with radioimmunoprecipitation, and 2 × SDS sample buffer was added to elute the biotinylated proteins for western blot analysis. Full-length blots are shown in Supplementary Fig. 9.

### Membrane fractionation

Mouse hippocampi were homogenized in 0.25 M sucrose, 10 mM Tris (pH 8), 1 mM MgCl_2_, 1 mM EDTA and 0.5 mM DTT with protease inhibitors. Lysates were centrifuged at 800*g* for 10 min at 4 °C to separate the nuclear fraction; the supernatant was then centrifuged at 5,000*g* for 10 min at 4 °C to separate the mitochondrial fraction and at 100,000*g* for 60 min at 4 °C to separate the membrane fraction. The final supernatant was the cytosolic fraction.

### Electrophysiology and stereotaxic surgery

Mouse and rat hippocampal neurons at 17–21 days *in vitro* were used to measure mEPSCs. For mEPSC recording, hippocampal cultures from WT and *nNOS*^−/−^ mice were used for p35 knockdown experiments, whereas rat hippocampal cultures were used for L-NAME treatment experiments.

VSV-G pseudotyped lentiviral particles with pFUGW-GFP were delivered into the mouse hippocampal CA1 region (anteroposterior: −2.00 mm, mediolateral: ±1.70 mm, dorsoventral: −1.4 mm, relative to the bregma) for 4 weeks using a Quintessential Stereotaxic Injector (Stoelting Company) with an injection volume of 3 μl at 0.2 μl min^−1^ through a customized ∼100-μm borosilicate glass microcapillary tip. After surgery, the animals were placed under a heating lamp until reawakening.

### Cell culture and plasmid transfection

HEK293T and COS7 cells were cultured in DMEM (Invitrogen) supplemented with 10% heat-inactivated fetal bovine serum plus antibiotics. Cells were transfected with expression constructs using Lipofectamine Plus reagent (Invitrogen). Primary hippocampal neurons and cortical neurons were prepared from embryonic day (E) 18–19 rat embryos or E18 mouse embryos, seeded on cultured plates coated with poly-D-lysine (50 μg ml^−1^) and maintained in Neurobasal medium supplemented with 2% B27 and 0.5 mM glutamine (Invitrogen). Cultured rat and mouse hippocampal neurons were used for morphological observations, surface receptor labelling and mEPSC recordings. Meanwhile, cultured rat cortical neurons were used for biochemical analyses. Hippocampal neurons at 12–14 days *in vitro* were transfected with different plasmids plus enhanced GFP using calcium phosphate precipitation. For drug treatment, 100 μM GSH/GSNO, 100 μM SNOC, 10 μM MG132, 10 μg ml^−1^ CHX, 1 mM DTT, 300 μM L-NAME, 100 μM NMDA or 25 μM roscovitine was added to the cultured neurons or cultured slices unless stated otherwise.

### Biotin switch assay

The biotin switch assay was performed to detect protein *S*-nitrosylation^54^. Briefly, adult mouse brains or HEK293T cells overexpressing p35 or its cysteine mutants were lysed in HENT buffer (250 mM HEPES, 1 mM EDTA, 0.1 mM Neocuproine, and 1% Triton X-100, pH 7.7). The cell lysates were then incubated with 10 mM *S*-methyl methanethiosulfonate for 20 min at 50 °C to block free cysteine, and excess *S*-methyl methanethiosulfonate was removed by three passes through a G25 Sephadex spin column. The samples were then incubated with 5 mM ascorbate to specifically reduce *S*-nitrosylated cysteine to free cysteine and 0.4 mM *N*-[6-(biotinamido)hexyl]-3′-(2′-pyridyldithio) propionamide at room temperature with rotation for 1 h to label the reduced cysteines. Unreacted *N*-[6-(biotinamido)hexyl]-3′-(2′-pyridyldithio) propionamide was removed by a G25 Sephadex spin column, and the biotinylated samples were then incubated with 50 μl NeutrAvidin agarose for 1 h. The pellets were washed five times with neutralization buffer with 0.6 M NaCl, eluted by SDS sample buffer and subjected to western blot analysis. Full-length blots are shown in Supplementary Fig. 9.

### *In vitro* kinase assay

Cdk5/p35 protein was precipitated from mouse hippocampal lysate or cultured rat cortical neuron lysate, pelleted by centrifugation and washed three times with lysis buffer. The Cdk5/p35 kinase assay was subsequently performed in kinase buffer (20 mM MOPS (pH 7.4) and 15 mM MgCl_2_) containing 200 μM ATP and 8 μg histone H1 protein for 30 min at 30 °C in a final volume of 30 μl. Phosphorylated histone H1 protein was then separated by 15% SDS-PAGE and blotted by anti-phospho-histone H1 antibody. The band intensity was quantified using Image J (National Institutes of Health, Bethesda). Full-length blots are shown in Supplementary Fig. 9.

### Statistical analysis

All data are presented as the mean±s.e.m. from at least three independent experiments. The significance of differences was determined by unpaired Student's *t*-tests or one-way analysis of variance with the Student–Newman–Keuls test. The level of significance was set at *P*<0.05. No samples or animals were excluded. All samples from each group were analysed to confirm a normal distribution and equal variance.

## Additional information

**How to cite this article:** Zhang, P. *et al.* S-nitrosylation-dependent proteasomal degradation restrains Cdk5 activity to regulate hippocampal synaptic strength. *Nat. Commun.* 6:8665 doi: 10.1038/ncomms9665 (2015).

## Supplementary Material

Supplementary Information Supplementary Figures 1-9 and Supplementary Table 1
