# Supplementary material for: S-nitrosylation-dependent proteasomal degradation restrains Cdk5 activity to regulate hippocampal synaptic strength
Source: Nat Commun. 2015 Oct 27;6:8665. doi: 10.1038/ncomms9665 (PMC4639907; doi:10.1038/ncomms9665)
Supplement: Supplementary Information — Supplementary Figures 1-9 and Supplementary Table 1 [file ncomms9665-s1.pdf]

SUPPLEMENTARY FIGURES

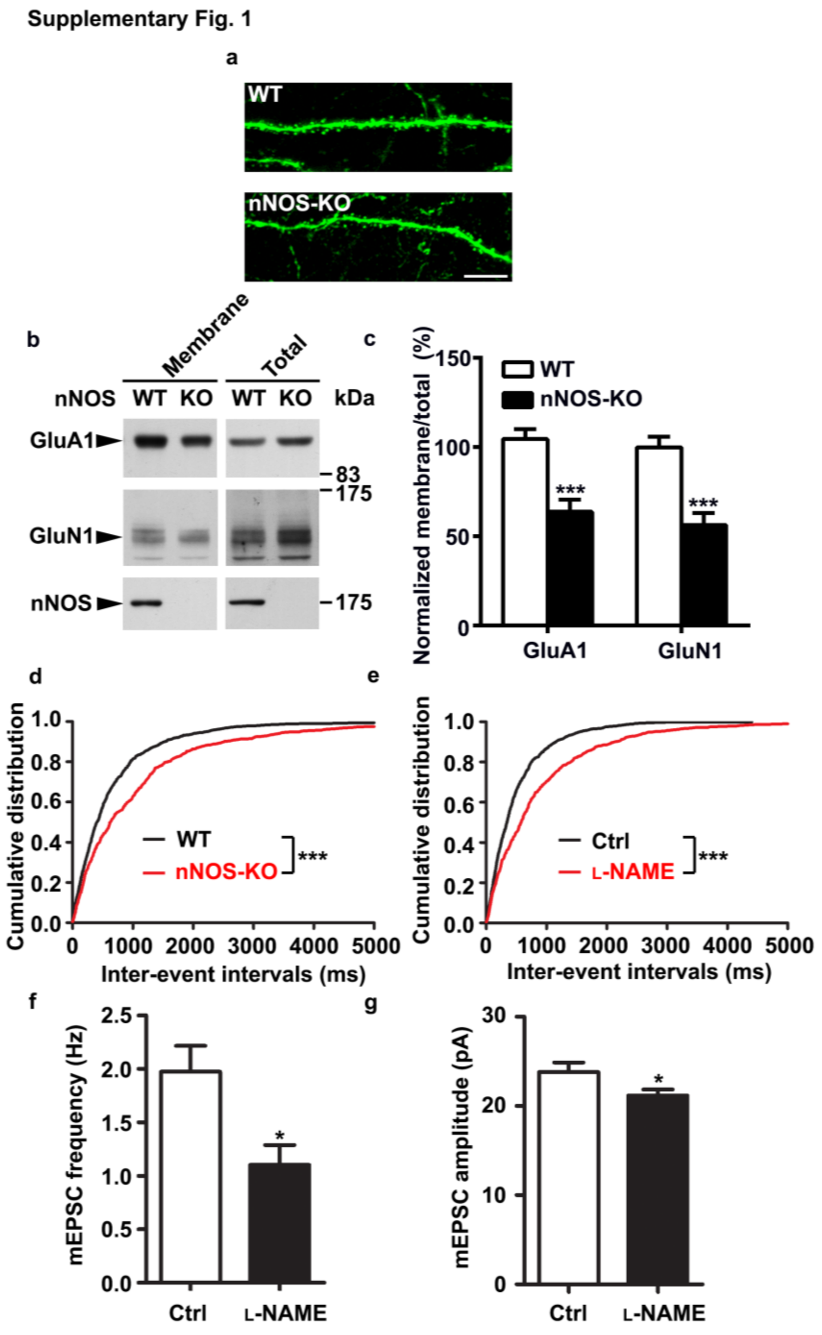

Supplementary Figure 1. Compromised synaptic function upon NO production blockade. (a) Representative images of dendritic spines visualized by immunostaining with anti-GFP antibody. Scale bar: 10 μm. (b, c) Neuronal nitric oxide synthase (nNOS)-knockout (KO) mice exhibited reduced GluA1 and GluN1 expressions in membrane fraction. (b) Hippocampi were prepared from 3-month-old

wild-type (WT) and nNOS-KO mice and subsequently subjected to membrane fractionation, followed by Western blot analysis with the indicated antibodies. (c) Quantification of proteins in membrane fractions (ratio of proteins in membrane fraction to the total level) in WT and nNOS-KO mice. Data are normalized to those of WT controls and represent the mean ± SEM of 4 independent experiments, $n = 6–8$ mice/condition. ***$p = 4.96E-4$ (GluA1), ***$p = 7.25E-4$ (GluN1); unpaired Student's *t*-test. (d) The miniature excitatory postsynaptic current (mEPSC) frequency was decreased in nNOS-KO hippocampal neurons. Cumulative distribution of inter-event intervals (inversely proportional to frequency) in cultured hippocampal neurons derived from WT and nNOS-KO mice (***$p = 3.56E-8$; Kolmogorov–Smirnov test). (e–g) L-NAME treatment decreased mEPSC frequency and amplitude in cultured rat hippocampal neurons. Cultured rat hippocampal neurons at 18–20 days *in vitro* (DIV) were treated with L-NAME for 4 h. (e) Cumulative distribution of inter-event intervals (inversely proportional to frequency; ***$p = 1.42E-8$; Kolmogorov–Smirnov test) and quantification of frequency (f) and amplitude (g) of mEPSCs. Data represent the mean ± SEM of 3 independent experiments, $n = 10–15$ neurons/condition; *$p = 0.018$ (frequency), *$p = 0.047$ (amplitude); unpaired Student's *t*-test.

Supplementary Fig. 2

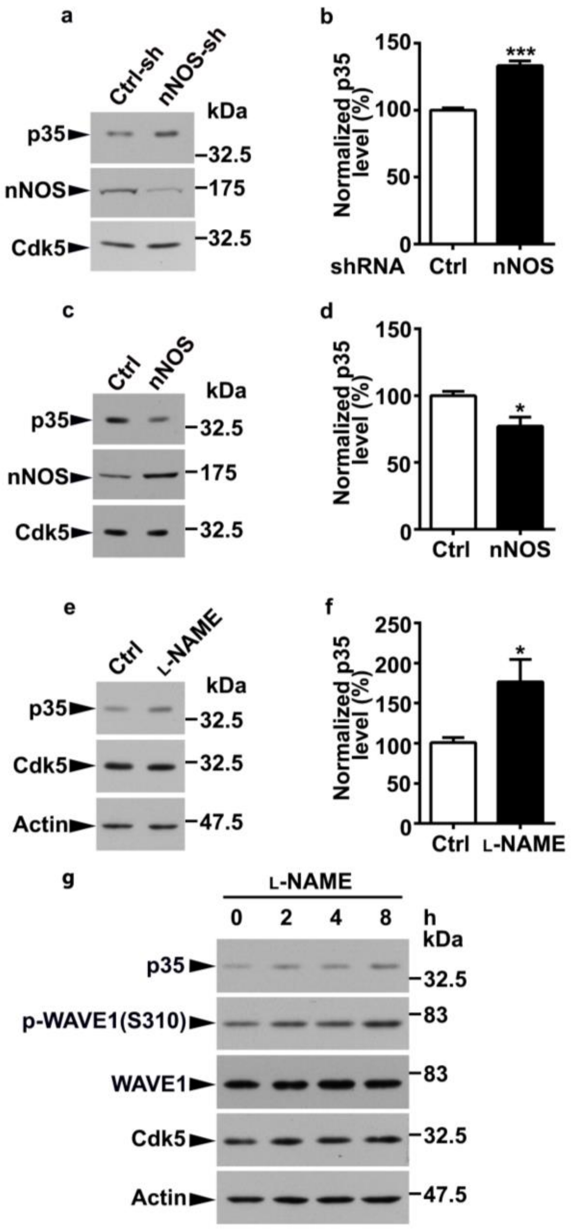

Supplementary Figure 2. NO signaling negatively regulates p35 level. (a, b) p35 was increased in nNOS-knockdown neurons. (a) nNOS was knocked down in cultured rat cortical neurons, and the cells were harvested and subjected to Western blot analysis using the indicated antibodies. (b) Quantification of p35 levels. Data represent the mean ± SEM of 5 independent experiments. ***p = 1.56E-4; unpaired Student's t-test. (c, d) Ectopic expression of nNOS decreased p35 level. (c) nNOS was

overexpressed in cultured rat cortical neurons, and the cells were harvested and subjected to Western blot analysis using the indicated antibodies. (d) Quantification of p35 levels. Data represent the mean ± SEM of 4 independent experiments. *p = 0.019; unpaired Student's *t*-test. (e, f) L-NAME treatment increased p35 level. (e) Cultured rat cortical neurons at 10 DIV were treated with L-NAME for 4 h and subjected to Western blot analysis with the indicated antibodies. (f) Quantification of p35 levels. Data represent the mean ± SEM of 4 independent experiments. *p = 0.039; unpaired Student's *t*-test. (b, d, f) Band intensities of p35 were normalized to the actin levels; and the results of the control group was set as 100%. (g) L-NAME treatment increased Cdk5-dependent phosphorylation of WAVE1. Cultured rat cortical neurons at 10 DIV were treated with L-NAME for the indicated times and subjected to Western blot analysis using the indicated antibodies.

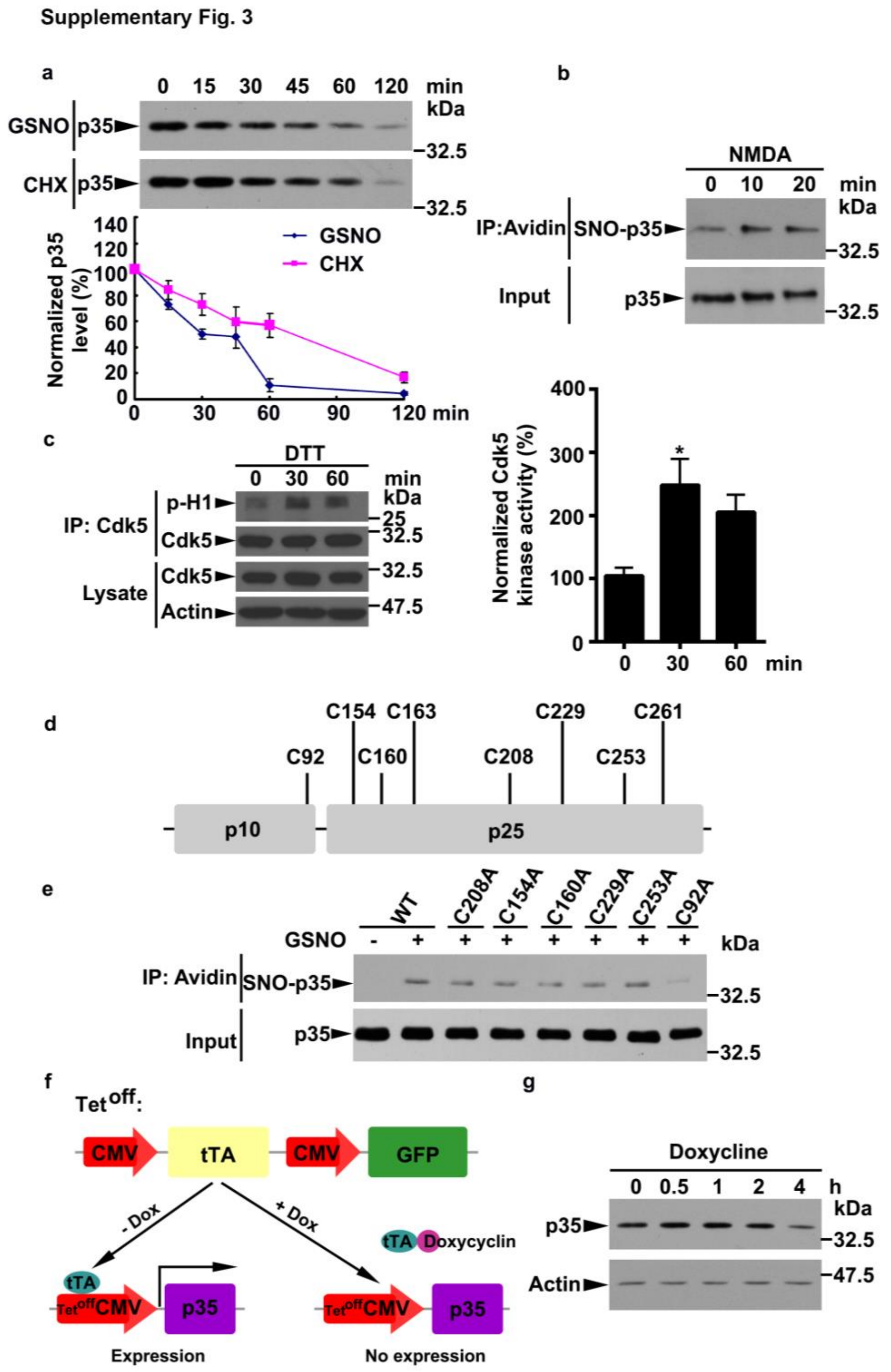

Supplementary Figure 3. S-nitrosylation of p35 promotes its degradation. (a) Cultured rat cortical neurons were treated with GSNO or cycloheximide (CHX) for the indicated times and then subjected to Western blot analysis. (b) NMDAR activation increases p35 S-nitrosylation. Cultured rat cortical neurons at 7-10 DIV

were treated with NMDA for the indicated times and subsequently subjected to the biotin switch assay. (c) DTT treatment enhances Cdk5 activity. Cultured rat cortical neurons at 7-10 DIV were treated with DTT for the indicated times and subsequently subjected to the *in vitro* kinase assay. Data are normalized to the control group and represent the mean ± SEM of 3 independent experiments. $*p = 0.035$; one-way ANOVA with the Student–Newman–Keuls test. (d) Schematic diagram showing the 8 potential S-nitrosylation sites on p35. (e) Cys92 is the target site for S-nitrosylation on p35. HEK293T cells were transfected with expression constructs encoding the WT or specific cysteine mutants of p35 as indicated. The cell lysate was then incubated with GSNO for 30 minutes and subjected to the biotin switch assay. The biotinylated proteins were immunoprecipitated (IP) with NeutrAvidin agarose followed by Western blot analysis for p35. (f) Schematic representation of the Tet^{OFF} system in which p35 expression is switched off upon the addition of doxycycline (Dox). (g) p35 was overexpressed in COS7 cells using the Tet^{OFF} system for 24 h, treated with Dox for the indicated times, and subjected to Western blot analysis using indicated antibodies.

Supplementary Fig. 4

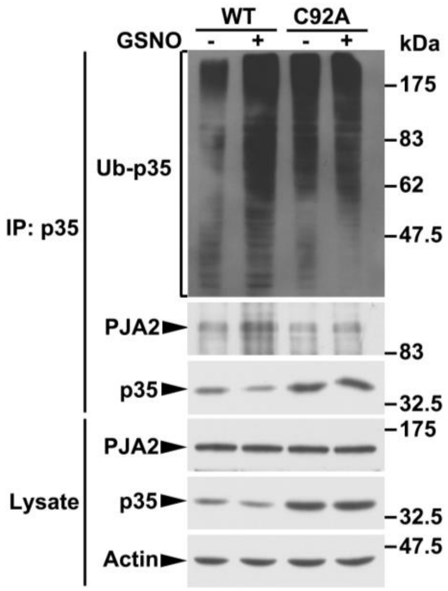

Supplementary Figure 4. p35 degradation is mediated through the E3 ubiquitin ligase PJA2. HEK293T cells were transfected with p35-WT or p35-C92A mutant; 24 h after transfection, the cells were treated with GSH/GSNO for 1 h, harvested, immunoprecipitated using p35 antibody, and subjected to Western blot analysis using the indicated antibodies.

Supplementary Fig. 5

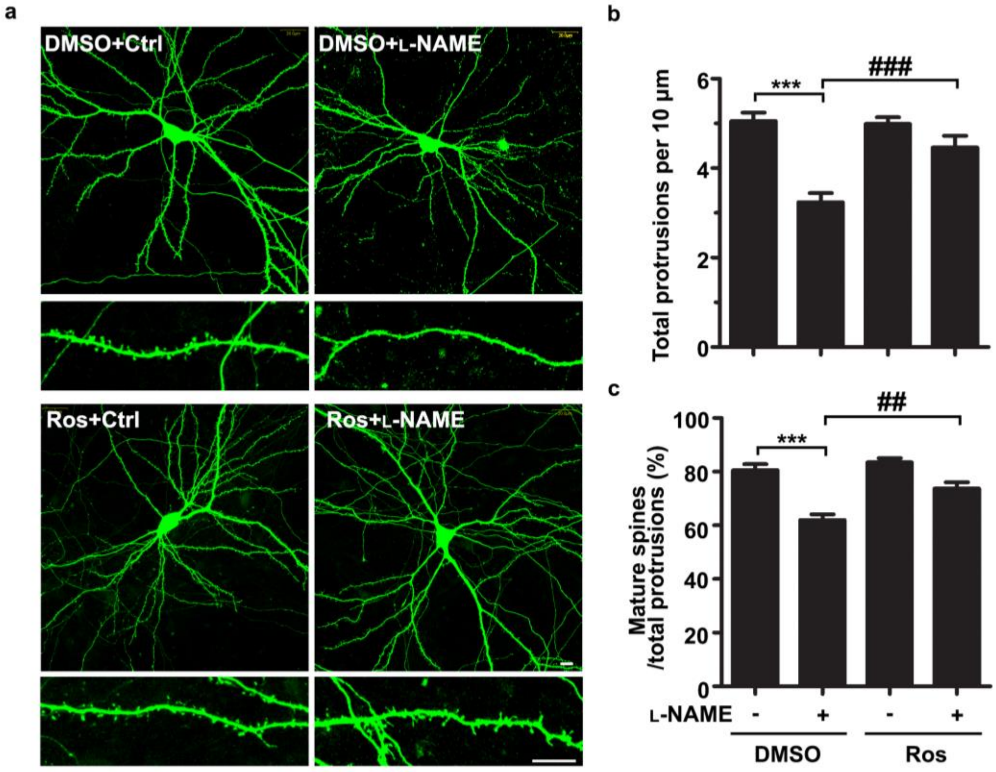

Supplementary Figure 5. NO signaling regulates dendritic spine density and morphology via the downregulation of Cdk5 activity. (a) Cultured rat hippocampal neurons were transfected with GFP construct at 12–13 DIV, and treated with L-NAME in the presence or absence of roscovitine (Ros) at 20 DIV for 8 h. Representative images are shown. Scale bar: 10 µm. (b) Quantification of spine density and (c) percentages of mature spines (i.e., spines with mushroom-shaped heads). Data represent the mean ± SEM of 3 independent experiments, n = 30–36 dendrites from 10–15 neurons/condition. ***p = 5.91E-8 (total protrusions), ***p = 4.67E-7 (mature spine proportion), $^{###}p$ = 6.43E-4, $^{##}p$ = 0.00595; one-way ANOVA with the Student–Newman–Keuls test.

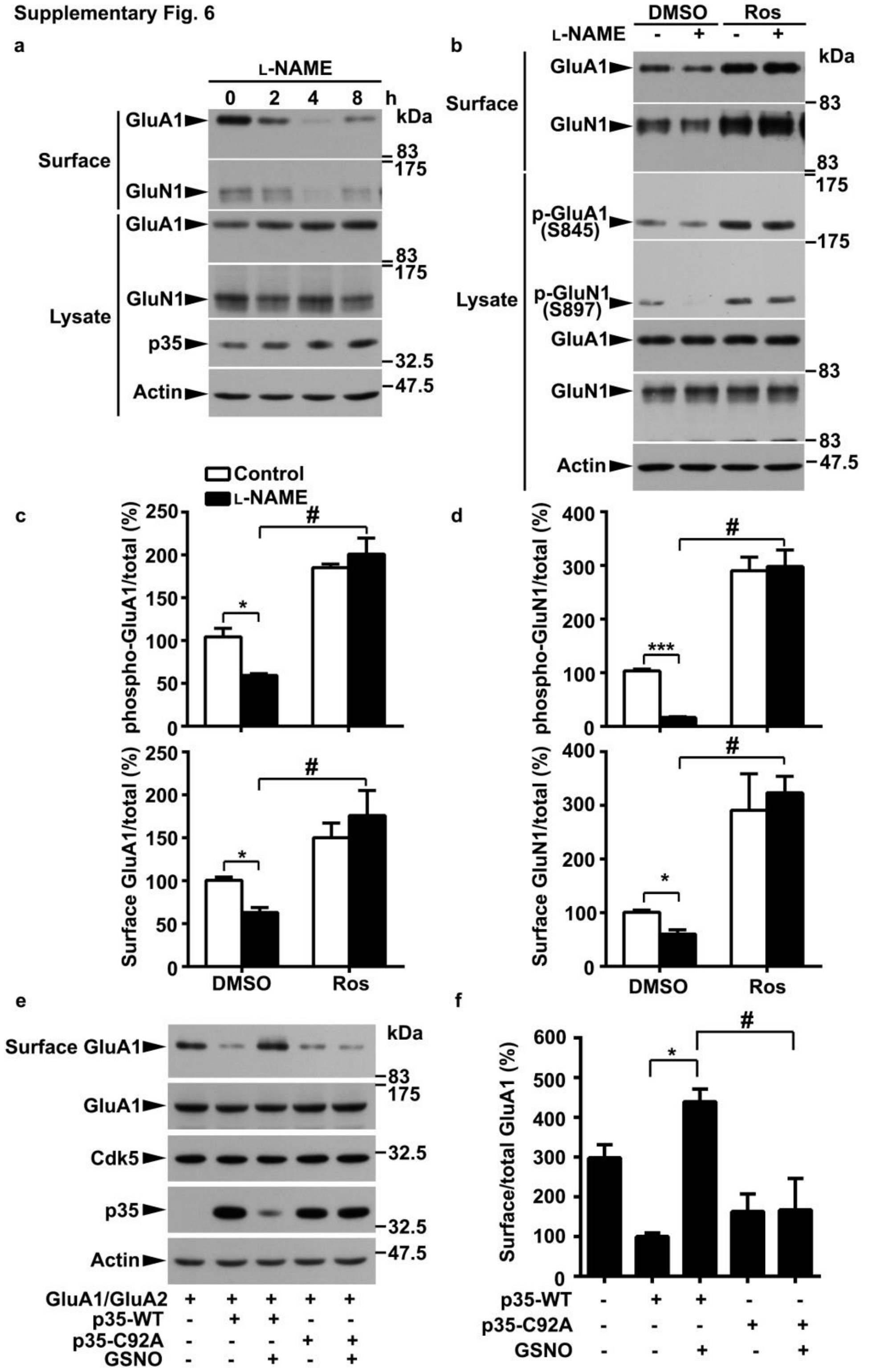

Supplementary Figure 6. p35 S-nitrosylation at Cys92 regulates GluA1 surface expression. (a) L-NAME treatment reduced surface levels of GluA1 and GluN1 in cultured rat hippocampal neurons. Rat hippocampal neurons at 17–18 DIV were

treated with L-NAME for the indicated times and subsequently subjected to the surface biotinylation assay. (b) Rat hippocampal neurons at 17–18 DIV were treated with L-NAME in the presence or absence of roscovitine (Ros) and subsequently subjected to the surface biotinylation assay. The biotinylated proteins were IP with streptavidin agarose followed by Western blot analysis with the indicated antibodies. (c, d) Quantification of phospho- and surface protein levels (ratio of phospho- and surface to total) in cultured rat hippocampal neurons. Data are normalized to those of the control group and represent the mean ± SEM of 6 independent experiments. Phospho-GluA1: $*p = 0.023$, $^{\#}p = 0.017$; phospho-GluN1: $***p = 0.00077$, $^{\#}p = 0.028$; surface GluA1: $*p = 0.031$, $^{\#}p = 0.029$; surface GluN1: $*p = 0.036$, $^{\#}p = 0.023$; one-way ANOVA with the Student–Newman–Keuls test. (e) p35 S-nitrosylation at Cys92 regulates GluA1 surface expression. HEK293T cells were transfected with p35-WT or p35-C92A construct together with other cDNA plasmids as indicated; the cells were subsequently treated with GSH/GSNO in the presence of doxycycline (Dox) and subjected to the surface biotinylation assay. (f) Quantification of surface GluA1 levels (relative to total) in HEK293T cells. Data represent the mean ± SEM of 5 independent experiments. The result of the singly overexpressing p35-WT group (lane 2) was set as 100%. $*p = 0.018$, $^{\#}p = 0.033$, one-way ANOVA with the Student–Newman–Keuls test.

Supplementary Fig. 7

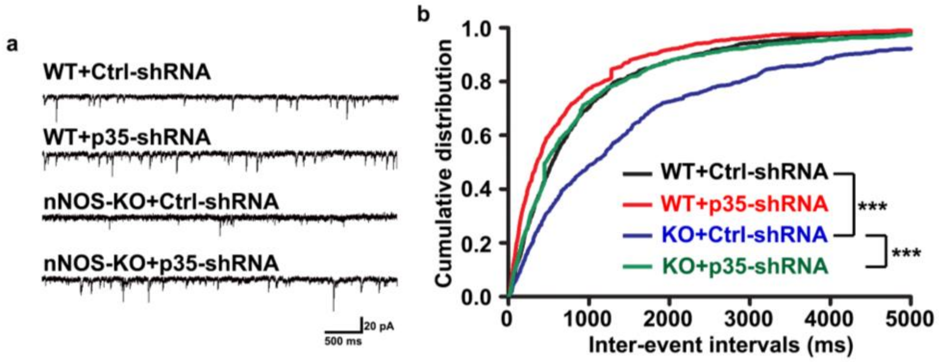

Supplementary Figure 7. Inhibition of Cdk5 activity rescues synaptic deficit upon NO production blockade. (a) Hippocampal neurons derived from WT or nNOS-KO mice were transfected with control (Ctrl)-shRNA or p35-shRNA together with GFP construct at 12–13 DIV, and mEPSCs were recorded at 20 DIV. Representative traces of electrophysiological recordings are shown. (b) Cumulative distribution of inter-event intervals (inversely proportional to frequency), ***p = 3.52E-9 (WT + Ctrl-shRNA vs KO + Ctrl-shRNA), ***p = 4.41E-8 (KO + Ctrl-shRNA vs KO + p35-shRNA); Kolmogorov–Smirnov test.

Supplementary Fig. 8

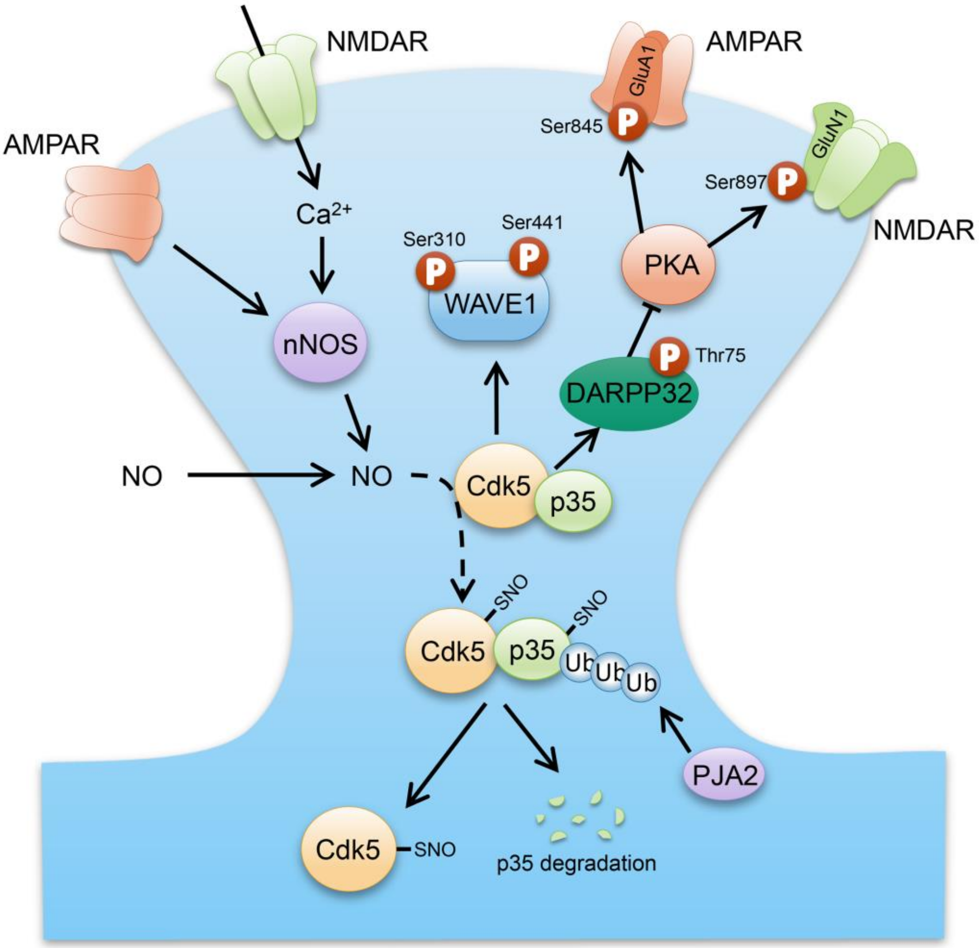

Supplementary Figure 8. Proposed working model of NO signaling actions at excitatory synapses via the regulation of Cdk5/p35. Nitric oxide (NO) signaling regulates dendritic spine morphogenesis as well as the surface expressions of AMPA receptor (AMPAR) subunit GluA1 and NMDA receptor (NMDAR) subunit GluN1 by p35 S-nitrosylation (p35-SNO) and promoting the ubiquitin proteasome-dependent degradation of p35 in a PJA2-dependent manner.

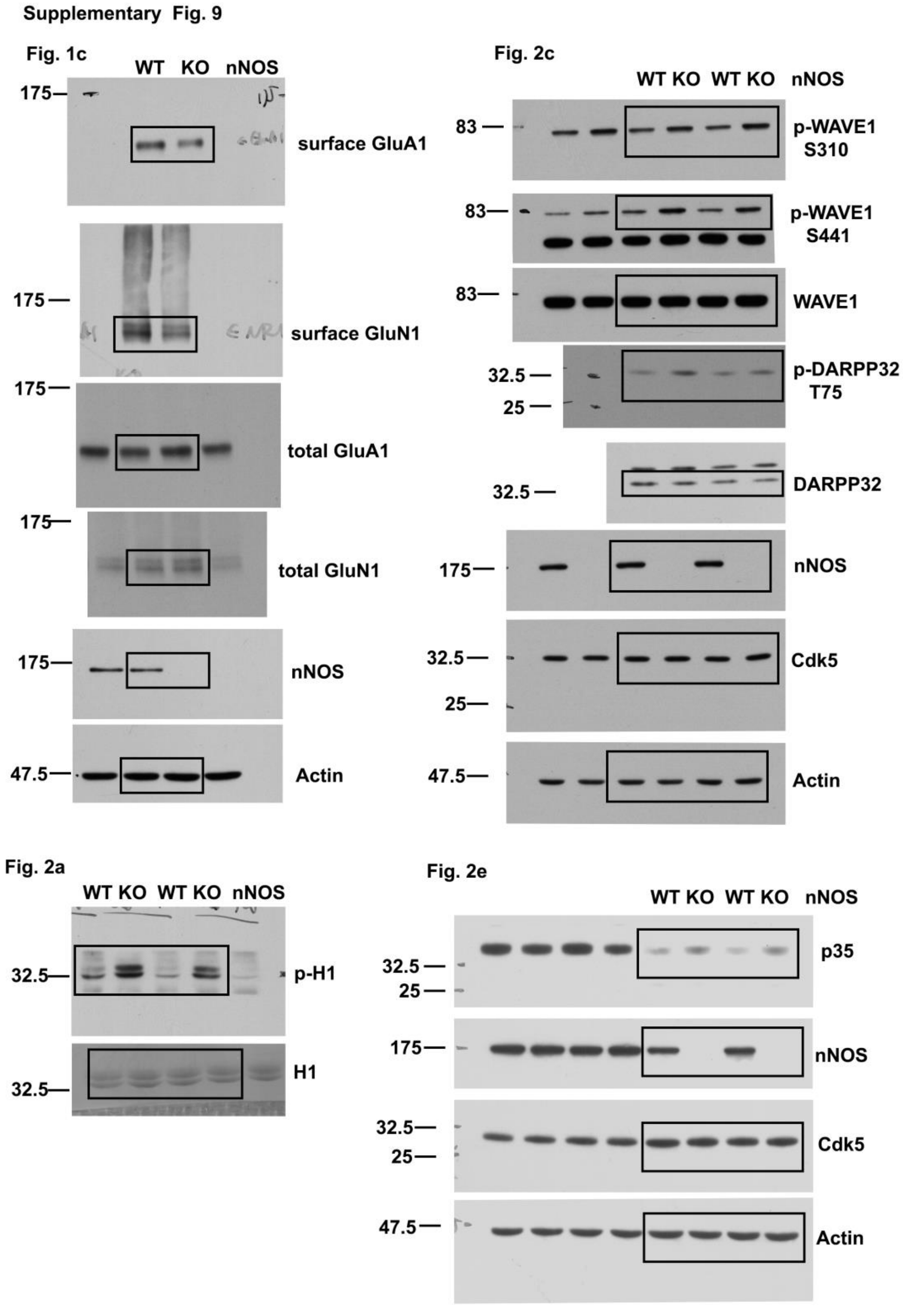

Supplementary Figure 9. Images of full-length blots presented in main Figures.

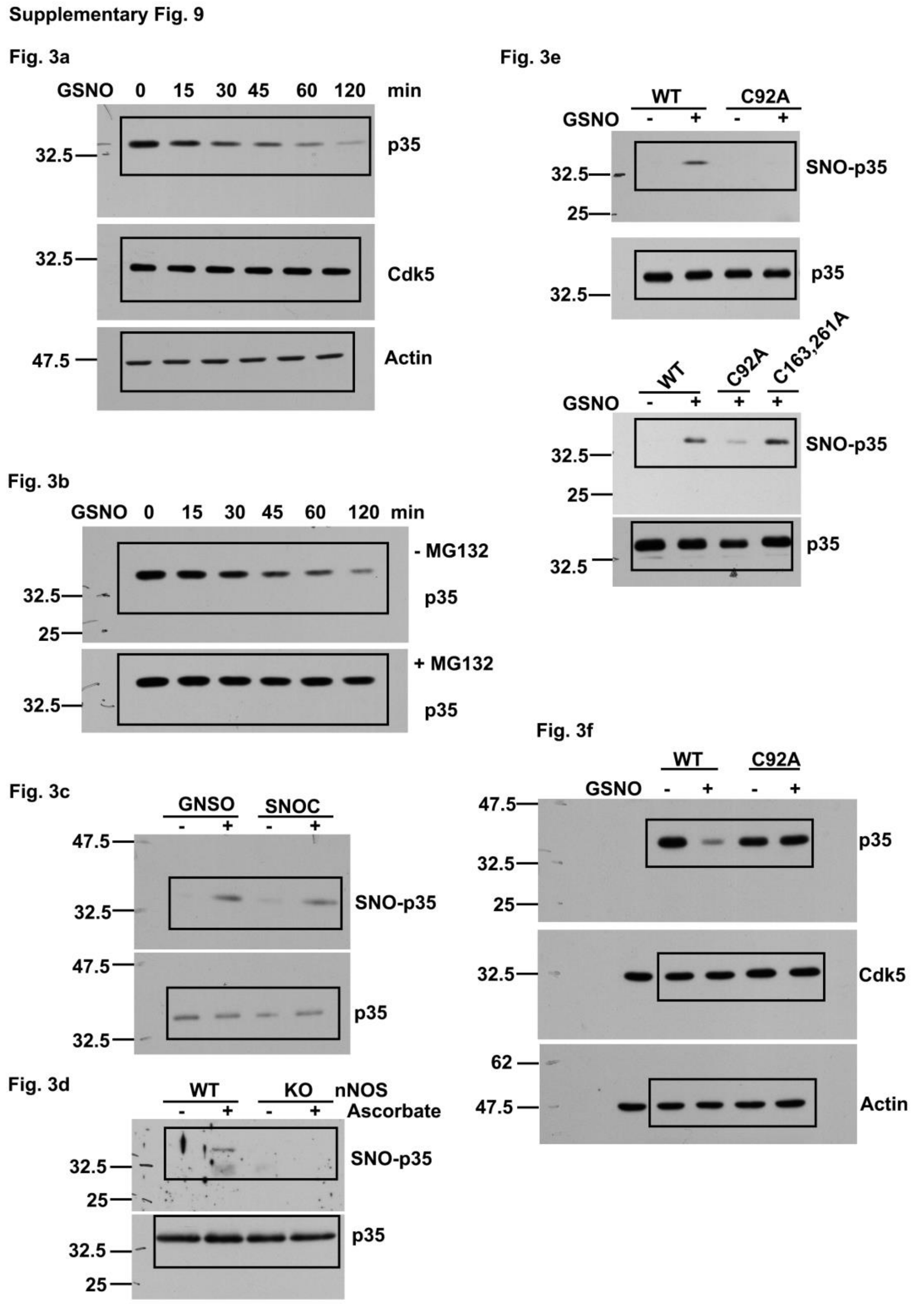

Supplementary Figure 9 (continued). Images of full-length blots presented in main Figures.

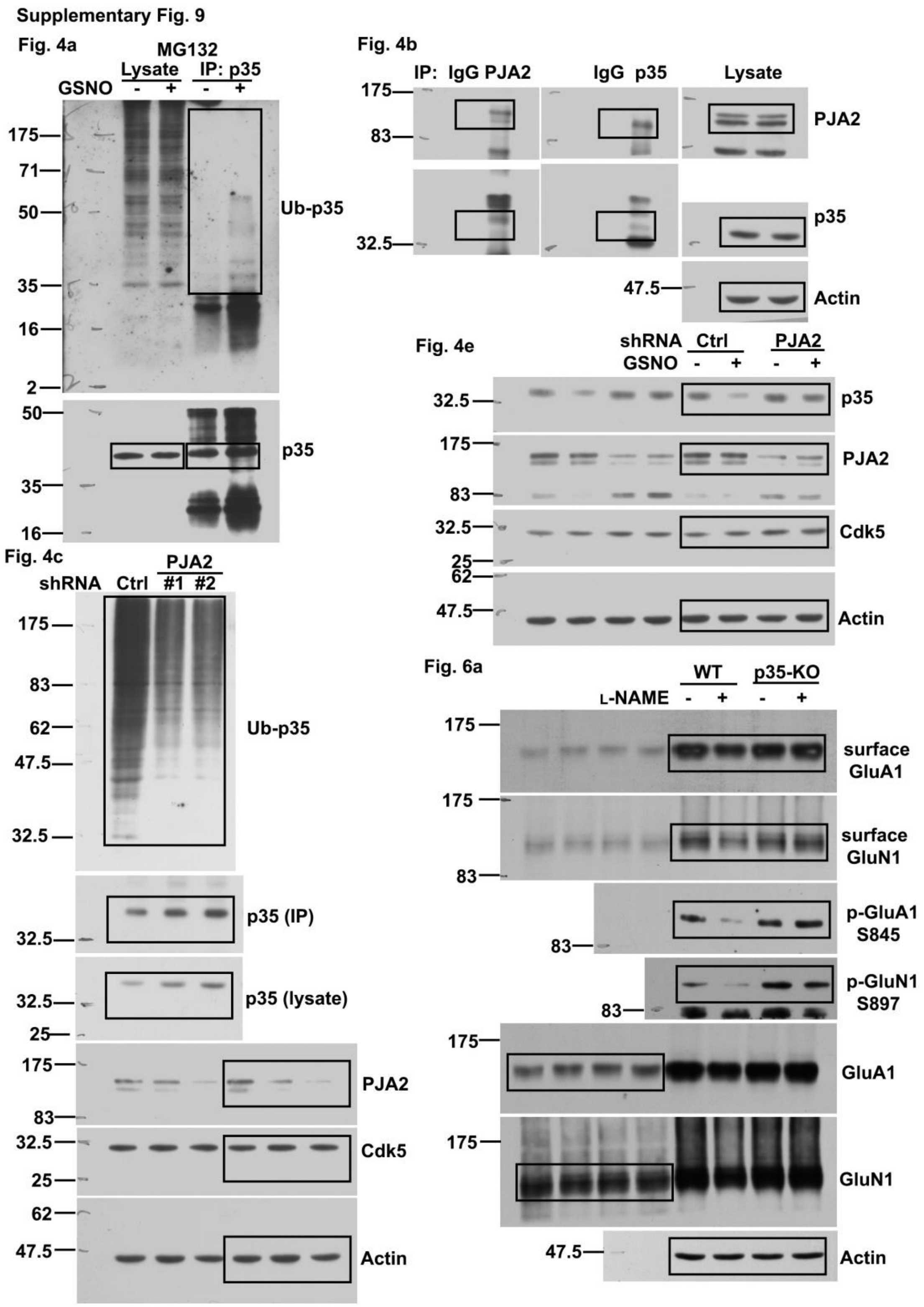

Supplementary Figure 9 (continued). Images of full-length blots presented in main Figures.

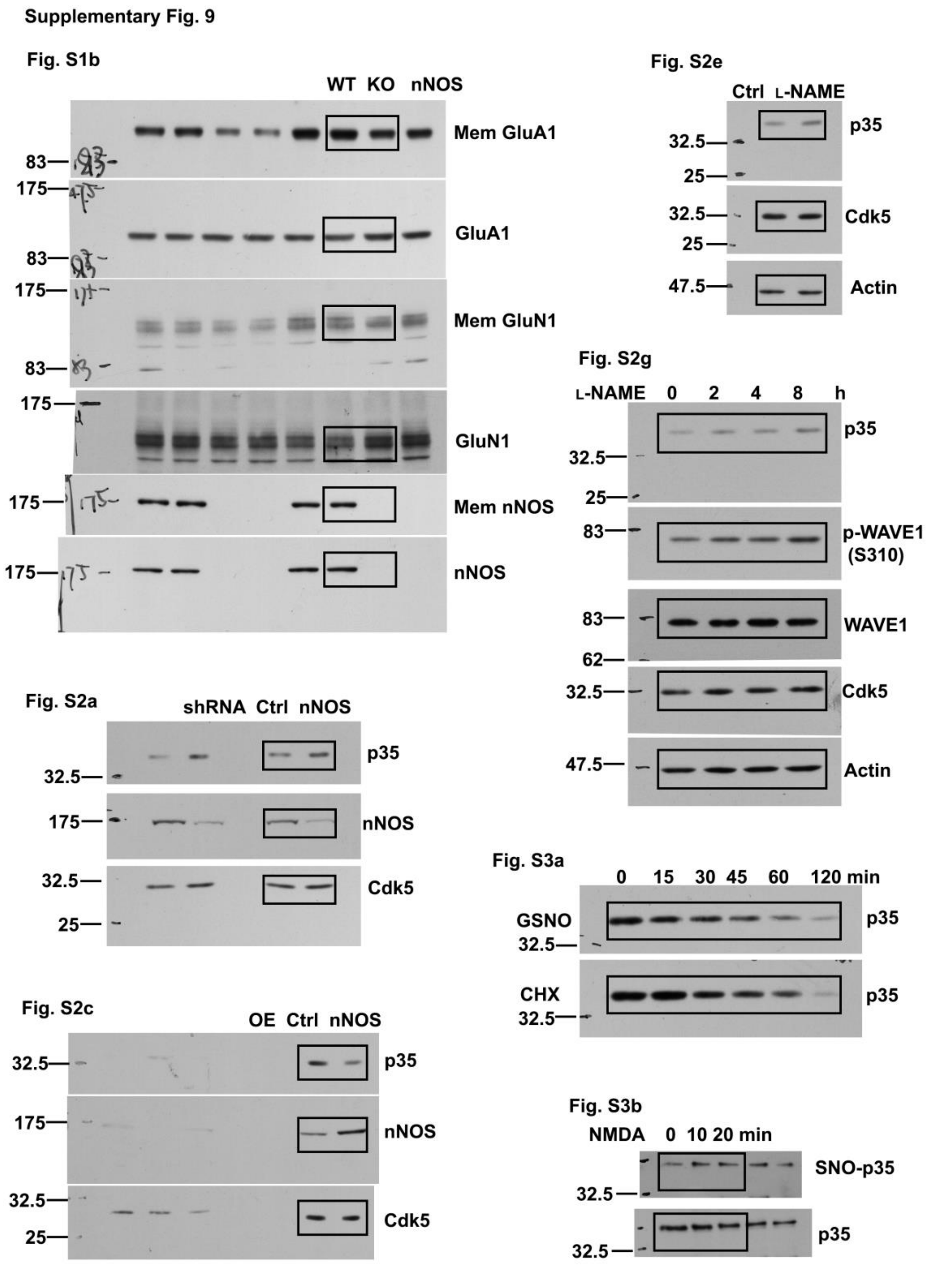

Supplementary Figure 9 (continued). Images of full-length blots presented in Supplementary Figures.

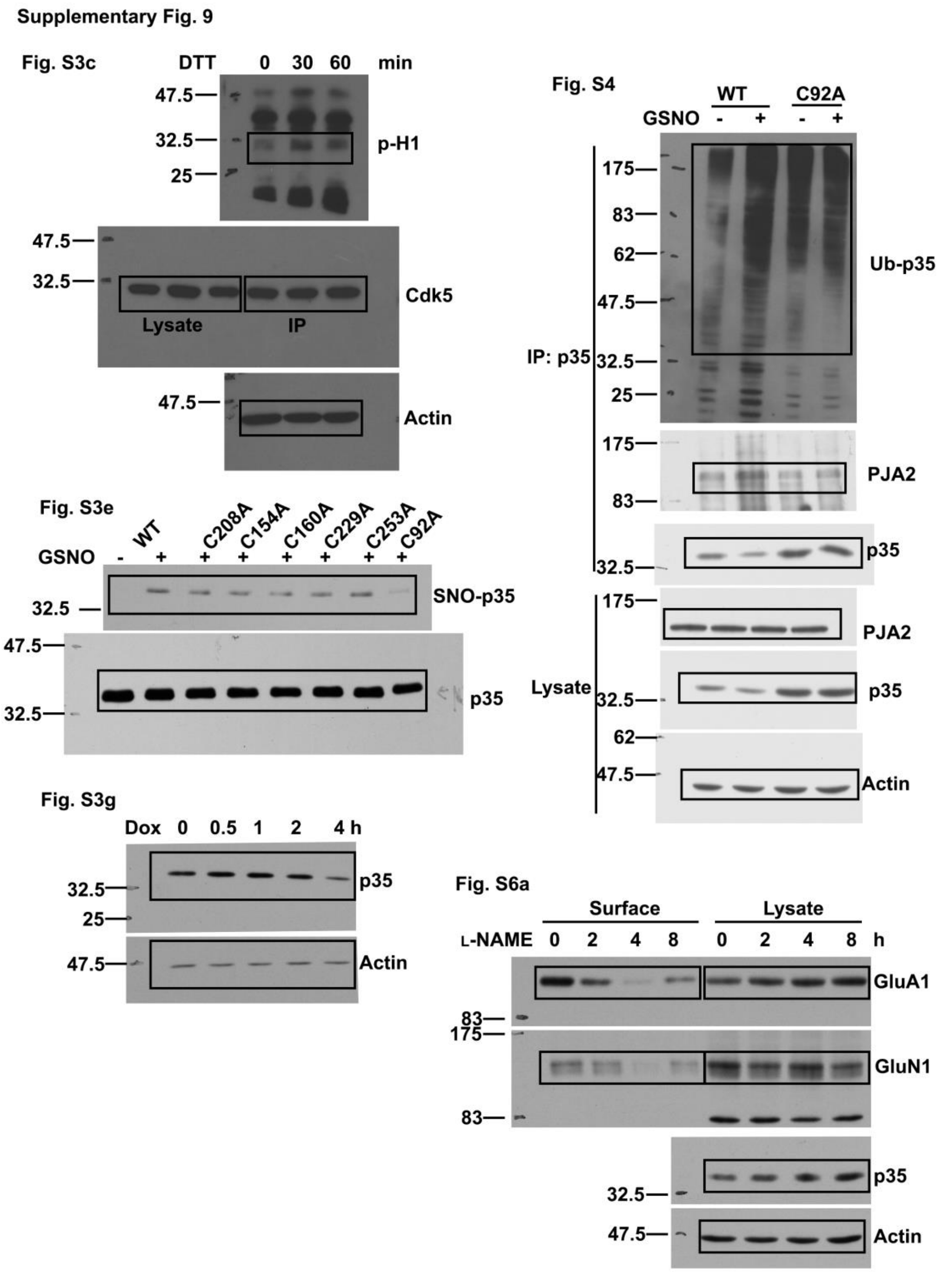

Supplementary Figure 9 (continued). Images of full-length blots presented in Supplementary Figures.

Supplementary Fig. 9

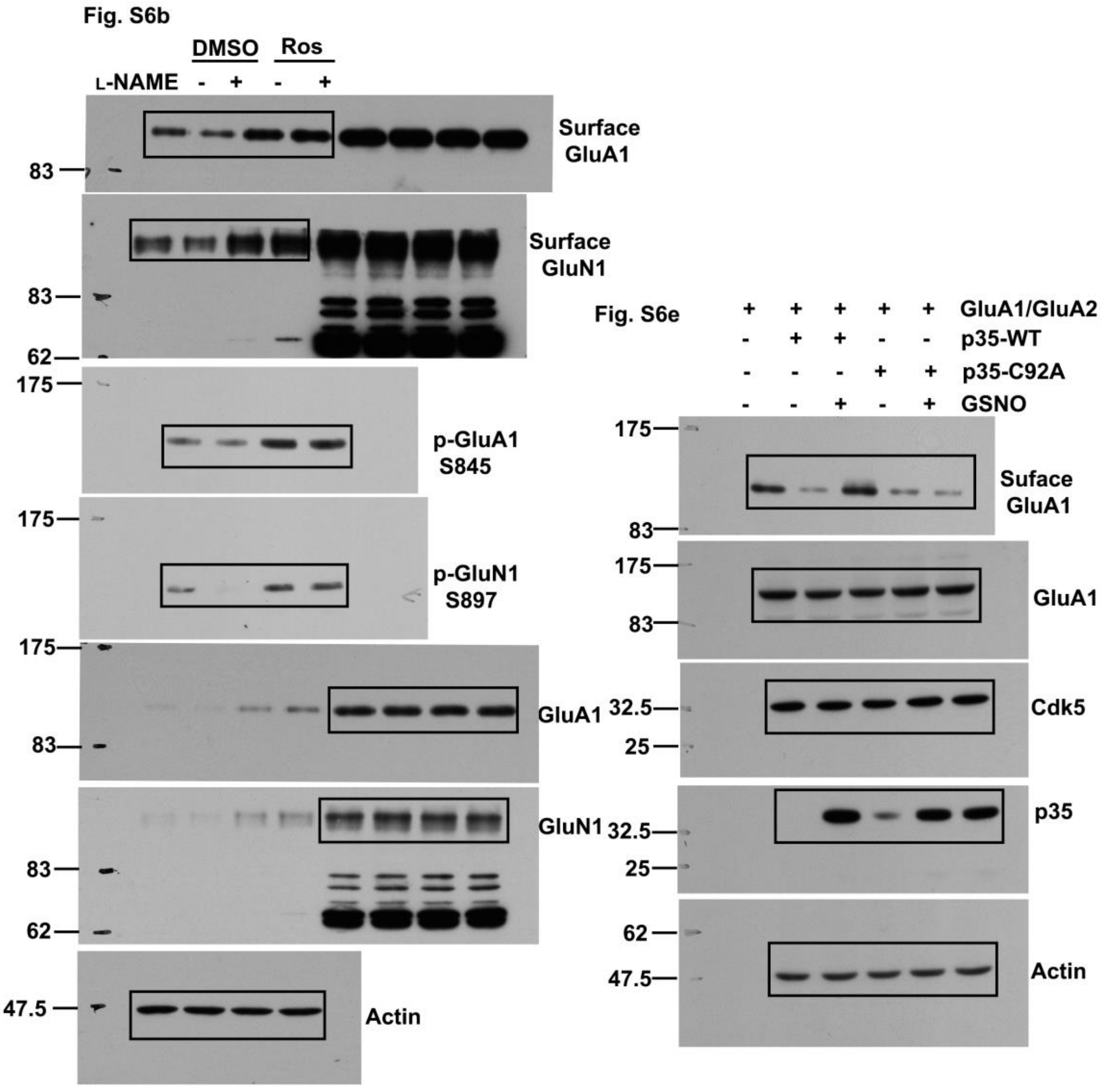

Supplementary Figure 9 (continued). Images of full-length blots presented in Supplementary Figures.

Supplementary Table 1: Primers for p35 S-nitrosylation mutants

Primer Name	Sequence
C92A F	5'-cctgaaaaaatcgctgtccgctgccaacctttccacattt-3'
C92A R	5'-aaatgtggaaaggttggcagcggacagcgatttttcagg-3'
C154A F	5'-gtgagctgctgcgcgccctgggtgagtttc-3'
C154A R	5'-gaaactcacccagggcgcgcagcagctcac-3'
C160A F	5'-ctgggtgagtttctcgcccgccggtgctacc-3'
C160A R	5'-ggtagcaccggcgggcgagaaactcacccag-3'
C163A F	5'-tctctgccgccgggcctaccgcctgaag-3'
C163A R	5'-cttcaggcggtaggcccggcggcagaga-3'
C208A F	5'-gtcttcctctacatgctcgccagggatgttatctcctc-3'
C208A R	5'-gaggagataacatccctggcgagcatgtagaggaagac-3'
C229A F	5'-ggctgtcctgctgaccgctctgtacctctcctat-3'
C229A R	5'-ataggagaggtacagagcggtcagcaggacagcc-3'
C253A F	5'-cttcctggtggagagcgccaaggaagccttttgg-3'
C253A R	5'-ccaaaaggcttccttggcgctctccaccaggaag-3'
C261A F	5'-ggaagccttttgggaccgtgccctctcagttatcaacc-3'
C261A R	5'-ggttgataactgagagggcacggtcccaaaaggcttcc-3'
